# Economic Evidence in Occupational Therapy: A Rapid Review

**DOI:** 10.1177/00084174241306983

**Published:** 2025-01-06

**Authors:** Andrew R. Freeman, Nadine Larivière, Judith Baillet, Rachel Beauchemin, Étienne Lavoie-Trudeau, Myriam Martel, Mégan St-François

**Keywords:** Advocacy, Economic evidence, Occupational therapy, Rapid review, Défense des droits et intérêts, données économiques, ergothérapie, revue rapide

## Abstract

**Background.** Given the necessity to demonstrate that occupational therapy services are a good use of resources, understanding the state of economic evidence is essential. **Purpose.** This article presents a *rapid review* of this evidence. **Method.** Relevant articles were identified using SCOPUS. Eligible studies included economic analyses of interventions that included occupational therapy and were published in English or French after 1999. The findings were synthesized and then appraised using the *Quality of Health Economic Studies* (QHES) template. **Results.** The 135 studies identified were conducted in 23 countries and most commonly: with adults/older adults; in home, inpatient, outpatient, and rehabilitation centre settings; with individuals with cerebrovascular accident and orthopaedic conditions. The specific occupational therapy role was specified in 60% of the studies. Approximately 50% of the investigations used a randomized controlled trial and a cost effectiveness analysis, and 40% used a societal economic perspective. The average QHES score was 74.4/100 (reasonable quality). **Implications.** This review has revealed areas of relative strength, some important gaps, and potential directions for future action. Economic evidence that specifically identifies the occupational therapy contribution must continue to be gathered. The profession should consider the strategic alignment of its economic research (e.g., home care) to maximize its impact.

## Résumé

**Description.** Vu la nécessité de démontrer que les services d’ergothérapie constituent une bonne utilisation des ressources, il est essentiel de comprendre l’état des données probantes de nature économique à ce sujet. **But.** Cet article présente une revue rapide de ces données probantes. Méthodologie. Les articles pertinents ont été identifiés à l’aide de Scopus. Pour être admissible, une étude devait présenter l’analyse économique d’une intervention impliquant l’ergothérapie et avoir été publiée en anglais ou en français après 1999. Les résultats ont été synthétisés puis évalués à l’aide du modèle Quality of Health Economic Studies (QHES). **Résultats.** Les 135 études identifiées ont été menées dans 23 pays et, le plus souvent : avec des adultes/personnes âgées; à domicile, auprès de personnes hospitalisées, en consultation externe ou en centre de réadaptation; avec des personnes ayant eu un accident vasculaire cérébral et des personnes ayant des troubles orthopédiques. Le rôle particulier de l’ergothérapie était précisé dans 60% des études. Environ 50% des études ont eu recours à un essai contrôlé randomisé et à une analyse coût-efficacité, et 40% ont eu recours à une perspective économique sociétale. Le score QHES moyen était de 74,4/100 (qualité raisonnable). **Conséquences.** Cette revue rapide a révélé des domaines de force relative, quelques lacunes importantes, et des orientations potentielles pour les actions futures. Il faut continuer de collecter les données économiques qui identifient spécifiquement la contribution de l’ergothérapie. La profession devrait envisager l’alignement stratégique de sa recherche économique (p. ex., soins à domicile) afin d’en maximiser l’impact.

## Introduction

Within the context of health care and related services in which considerable competition exists for available resources (Anesi & Kerlin, 2021), not only is it necessary to demonstrate that occupational therapy services make a difference but that they also are a good use of available financial resources (World Federation of Occupational Therapists, 2016). The need for the profession to develop its capacity in this area is well recognized (Lambert et al., 2014). Successfully advancing this capacity involves, among other elements, being competent consumers of economic research evidence (World Federation of Occupational Therapists, 2021). Understanding the current state of this evidence is essential.

Various efforts have been made to review the economic evidence for occupational therapy services. Green and Lambert (2017), in their systematic review of health economic evaluations in occupational therapy, concluded that the nine papers appraised varied considerably in quality, the main concerns being the quality of the original clinical study, the statement of cost-perspective and time horizon, the choice of outcome units, the presentation of included costs, and the use and reporting of uncertainty analyses. Barras (2005), in her systematic review of occupational therapy home assessments on a range of outcome measures, concluded that low numbers of high level or high quality publications directly relevant to the effectiveness of occupational therapy home assessment and discharge planning were identified. Johanson et al. (2023), in their systematic review of occupational therapy return-to-work interventions for people with mental health disorders, concluded that in practice settings where individual placement and support was used, they were cost-effective in several contexts, while three studies showed larger effects and higher costs. An occupational therapy intervention added to treatment for major depression was indicated to be cost-beneficial and an advanced supported employment was cost-saving. The methodological quality varied considerably between studies. In Nagayama and colleagues’ (2016) systematic review, they concluded that occupational therapy for older people was clinically effective and cost-effective in comparison with standard care or other therapies. These intervention studies suggested potentially cost-effective means to motivate clients to maintain their own health. The authors also noted the limitations of the findings because of the high heterogeneity of the reviewed studies on full economic evaluations of occupational therapy for older people. Rahja et al. (2018), in their systematic review of occupational therapy approaches for people with cognitive and/or functional decline, concluded that structured interventions comprising multiple consultations and engaged caregivers delivered better functional and economic outcomes. Finally, Wales et al. (2022), in their systematic review of occupational therapy services for adults in acute and subacute care settings, concluded that occupational therapy for adults poststroke and post-traumatic brain injury, acute discharge planning, and pre- and post-hip replacement is cost-effective, although further research is needed to substantiate these findings.

Despite the important contribution made by these reviews regarding the economic evidence for some sectors of occupational therapy practice, they do not permit a global picture of the evidence in the profession. The significant ongoing resource-related pressures being experienced in the health care and related sectors suggest that decision makers (e.g., managers and ministry of health) require timely access to economic evidence in practical formats. To respond to this need, *rapid reviews* can be highly useful; they are a “…form of knowledge synthesis that accelerates the process of conducting a traditional systematic review through streamlining or omitting various methods to produce evidence for stakeholders in a resource-efficient manner” (Hamel et al., 2021, p. 80). This approach to summarizing the existing state of knowledge has emerged as an efficient tool to transmit evidence to decision makers more quickly (Garritty et al., 2021), including for occupational therapy services (MacPherson et al., 2023). The purpose of this article is to present a rapid review of the economic evidence for occupational therapy services across all practice domains. This approach is appropriate for strategically aligning the profession's progress in this area with decision makers’ imperatives (Langlois et al., 2019).

## Method

Although best practice guidelines for rapid reviews continue to evolve, the *Cochrane Rapid Reviews Methods Group* (Garritty et al., 2021) has proposed interim recommendations. This review has adhered to these recommendations.

### Setting the Research Question

This review is part of a multipronged capacity-building project involving the first two authors and the Canadian Association of Occupational Therapists (CAOT), the aim of which is to create deliverable knowledge translation tools regarding economic evidence for occupational therapy services that inform stakeholders within and outside the profession. The research question emerged over the course of the two first authors’ long-term involvement in building the profession's economic evidence capacity (e.g., Freeman & Larivière, 2018), their collaboration with CAOT, and their supervision of a group of occupational therapy students. The main question guiding the rapid review was: What is the economic evidence for occupational therapy services?

### Setting Eligibility Criteria

Articles were included according to the following criteria: (1) date of publication from the year 2000 onwards. Given the significant evolution that has occurred in occupational therapy practice, and the strategic orientation of a rapid review, the evidence during this period would be more likely to have contemporary relevance; (2) presence of an occupational therapy intervention, whether alone or in a multidisciplinary setting; (3) presence of an economic analysis, regardless of the type of analysis; (4) published in English and French, considering the authors’ mastery of both languages; and (5) empirical studies. Articles were excluded based on the following criteria: (1) non-human rehabilitation (e.g., animal rehabilitation); (2) research protocols for which the results had not yet been published; and (3) systematic reviews.

### Searching

The initial article search was conducted in January 2022 via the SCOPUS database, and updated in December 2023. This database was used given its extensive coverage of fields aligned with the research topic (UNC University Libraries, 2023). The search was carried out by the students under the supervision of the first two authors and following a librarian's advice. The syntax used to locate the relevant literature was the following: (TITLE-ABS-KEY(”occupational therap*” OR OT OR rehab* OR “occupational intervention*”)) AND (TITLE-ABS-KEY(economic*)) AND PUBYEAR > 2000 AND (LIMIT-TO (SUBJAREA,"MEDI”) OR LIMIT-TO (SUBJAREA,"HEAL”) OR LIMIT-TO (SUBJAREA,"NURS”) OR LIMIT-TO (SUBJAREA,"SOCI”) OR LIMIT-TO (SUBJAREA,"ECON”) OR LIMIT-TO (SUBJAREA,"PSYC”) OR LIMIT-TO (SUBJAREA,"NEUR”)) AND (LIMIT-TO (LANGUAGE,"English”) OR LIMIT-TO (LANGUAGE,"French”)).

### Study Selection

The article selection process took place in February 2022 (updated December 2023). The references for the initially identified articles were uploaded into the reference software *Zotero* (Corporation for Digital Scholarship, 2023) to eliminate duplicates. These references were then uploaded into the screening and data extraction software tool *Covidence* (Veritas Health Innovation Ltd., 2023) to facilitate the selection process and to generate a PRISMA flow chart. The students and the second author screened the titles and abstracts in duos working independently to make an initial selection of relevant articles for analysis. When differences arose regarding the inclusion of an article, these were resolved in dyad. When doubts arose about the inclusion of an article within the duos, the first two authors participated in the decision-making process. Following this step, the retained articles were then read in full and screened by pairs reviewing independently; in cases of conflict, a third reviewer examined the article.

### Data Extraction

Data extraction took place between May 2022 and April 2023 (updated December 2023), and was conducted by the students using an analysis template that was developed by the first two authors in collaboration with CAOT representatives working in the practice standards and advocacy areas. This template included the following sections: (1) article reference; (2) key study elements (age group, practice setting, health condition, primary occupational challenge, occupational therapy intervention evaluated, research design, type of economic analysis); (3) country where the research took place; (4) study objectives; (5) description of participants; (6) nature of the occupational therapy intervention; (7) main methodological elements; (8) main results; (9) main limitations of the study; and (10) *Quality of Health Economic Studies* (QHES) score (Ofman et al., 2003).

### Risk of Bias Assessment

The QHES was used to assess the quality of economic studies. It provides a “weighted, easy-to-use scoring system for assessing the quality of health economic evaluations” (Chiou et al., 2003, p. 33), and includes 16 items to be evaluated to obtain a score out of 100 points (although formal parameters for interpreting the scores were not provided, higher score corresponds to better quality). Two item examples include the following: “Were the perspective of the analysis (societal, third-party payer, etc.) and reasons for its selection stated?”; “Was incremental analysis performed between alternatives for resources and costs?” Inter-rater reliability for this instrument was reported as 0.81 (95% confidence interval) (Au et al., 2008); furthermore, the largest source of variation in the scores assigned to each article reviewed was systematic differences in the quality of the articles themselves, representing 56% of the variance. The procedure used by the authors followed Garritty and colleagues’ (2021) recommendations for rapid reviews and Gerkens and colleagues’ (2008) recommendations regarding assessment of the quality of economic evaluations. Namely, the article analysis template was pilot tested by the first two authors with five articles. Precision regarding each item of the template and the QHES was ensured; as necessary, additional source articles (e.g., randomized controlled trials published prior to the published economic analysis) were consulted. The students initially analyzed two articles each; these analyses were subsequently validated by a second reviewer (first two authors or a second student). All articles were then analyzed by dyads of two students, each rated individually and then through a consensus. Finally, all of the analyses were verified by the first or second author.

### Synthesis

The data from the template analyses, the components of which are specified in the Data Extraction section above, were entered in an Excel file. Descriptive statistics (e.g., frequencies and percentages) were calculated for the various components included in the analysis template.

### Ethics

Ethical approval was not necessary to conduct this rapid review.

## Results

### Search Results

**Figure 1.**
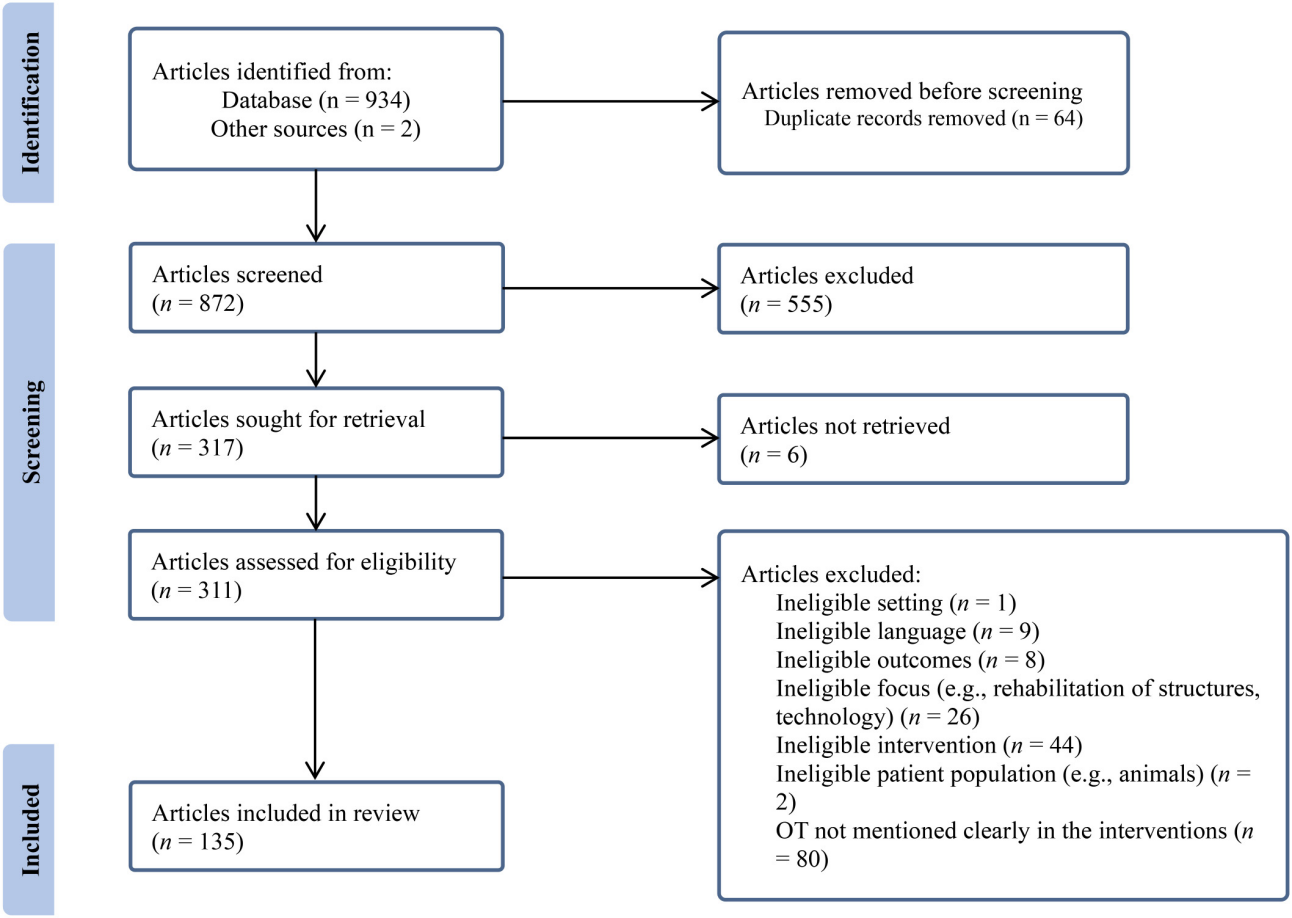
Flow of articles through the rapid review process.

The search result details are summarized in the PRISMA flow chart (Figure 1). The search revealed 872 articles once duplicates had been accounted for; 561 studies were excluded, leaving 311 articles for full review, which included two other articles identified in systematic reviews. Subsequently, 176 studies were excluded, leaving 135 studies to be included.

### General Description

Occupational therapy was the sole intervention investigated in 36 (26.7%) studies. Details regarding this intervention, whether within these investigations or in studies in which occupational therapy was part of the overall intervention, were provided in 80 (59.3%) investigations. Consistent with our rapid review orientation, we included all 135 studies in this analysis because of this finding's important implications for the profession. A summary of the articles is available as Supplemental Material.

The studies were conducted in 23 countries; 57 (42.3%) studies were conducted in two countries (United Kingdom, United States) and 108 (80.0%) were conducted in seven countries (Australia, Canada, Japan, Netherlands, Sweden, United Kingdom, United States). One hundred and eight (80.0%) of the studies included older adults (≥65 years) and 83 (61.5%) studies included adults (18–64 years). Seven (5.2%) and four (3.0%) investigations respectively included adolescents (13–17 years) and children (birth–12 years). Sixty-two (45.9%) studies included more than one age group, most commonly adults and older adults. One study (0.7%) was conducted with entry-level occupational therapy students.

### Practice Setting

Table 1 presents the practice settings in which the interventions being evaluated were conducted. In those studies in which the intervention was being compared with another intervention (control group), the key word (or words) we attributed for the practice setting was only for the intervention being tested. Given the different ways that services are organized in different countries and the variable terminology used, we could not always be certain about the comparability of practice settings across countries. The interventions in 55 (40.7%) of the studies were carried out at least in part in individuals’ homes; with minor exception, these investigations involved older adults. The interventions in 34 (25.2%) studies were carried out in more than one setting (e.g., outpatient and workplace).

**Table 1:** Practice Settings

Practice setting	Frequency (%)
Home	55 (40.7)
Inpatient^a^	36 (26.7)
Outpatient^b^	30 (22.2)
Rehabilitation centre^c^	24 (17.8)
Primary care clinic^d^	8 (5.9)
Nursing home^e^	6 (4.4)
Workplace	4 (3.0)
Community organization	3 (2.2)
Telerehabilitation	2 (1.5)
Emergency department	1 (0.7)
Post-secondary educational institution	1 (0.7)
Not specified	3 (2.2)

^a^ Inpatient refers to inpatient hospital. The distinction with inpatient rehabilitation centres is not always apparent.

^b^ Outpatient includes: outpatient, day hospital; day treatment centre; private clinic. It does not include rehabilitation centres.

^c^ Rehabilitation centre can include both inpatient and outpatient contact.

^d^ Primary care clinic includes: primary care clinics; community health centres.

^e^ Nursing home includes: nursing homes; long-term care.

### Conditions for Which Interventions Were Evaluated

The conditions experienced by individuals or groups for whom the interventions were evaluated are reported in Table 2. The most common condition was cerebrovascular accident (37; 27.6%). Thirteen (9.6%) studies included participants with more than one condition. In 23 (17.2%) investigations, the condition was not specified.

**Table 2:** Conditions

Condition	Frequency (%^a^)
Cerebrovascular accident	37 (27.6)
Orthopaedic^b^	13 (9.7)
Cognitive decline^c^	11 (8.2)
Frailty	9 (6.7)
Cardiovascular problems^d^	9 (6.7)
Pain^e^	8 (6.7)
Mental health condition^f^	6 (4.5)
Pulmonary disease^g^	6 (4.5)
Arthritis	5 (3.7)
Brain injury	5 (3.7)
Mixed neurological conditions	4 (3.0)
Chronic illness^h^	3 (2.2)
Parkinson's disease	3 (2.2)
Disability (not specified)	2 (1.5)
Obesity	2 (1.5)
Significant vision challenges^i^	2 (1.5)
Spinal cord injury	2 (1.5)
Autism spectrum disorder	1 (0.8)
Cerebral palsy	1 (0.8)
Fieldwork	1 (0.8)
Hand problem	1 (0.8)
Huntington disease	1 (0.7)
Musculoskeletal condition	1 (0.7)
Not specified	23 (17.2)

^a^ Based on 134 vs. 135 studies because Gospodarevskaya et al.'s (2019) study conducted with occupational therapy students. [Gospodarevskaya, E., Carter, R., Imms, C., Yee Chu, E. M., Nicola-Richmond, K. M., Gribble, N., Froude, E., Guinea, S., Sheppard, L., Iezzi, A., & Chen, G. (2019). Economic evaluation of simulated and traditional clinical placements in occupational therapy education. *Australian Occupational Therapy Journal*, *66*(3), 369–379].

^b^ Orthopaedic includes: orthopaedic; hip fracture; joint replacement surgery.

^c^ Cognitive decline includes: cognitive decline; dementia.

^d^ Cardiovascular problems include: cardiovascular problems; hypertension.

^e^ Pain includes: back pain; chronic pain; complex regional pain syndrome.

^f^ Mental health condition includes: serious mental illness; depression; emotional and behavioural disorders; panic disorder.

^f^ Pulmonary disease includes: chronic obstructive pulmonary disease; pneumonia; pulmonary disease.

^h^ Chronic illness includes: chronic illness (not specified); diabetes.

^i^ Significant vision challenges includes: glaucoma; low vision.

### Main Occupational Challenge

The occupational challenge being experienced by the participants is reported in Table 3. Although functional difficulties were the most frequently mentioned occupational challenge (90; 67.2%), its precise nature was not always clearly identified. Sixty-eight (50.7%) studies included more than one challenge. The occupational challenge was not specified in five (3.7%) studies.

**Table 3:** Main Occupational Challenge

Occupational challenge	Frequency (%^a^)
Functional difficulties^b^	90 (67.2)
Activities of daily living difficulties^c^	50 (37.3)
Quality of life	33 (24.6)
Falls risk	16 (11.9)
Mobility	15 (11.2)
Job tenure^d^	4 (3.0)
Caregiver burden	7 (5.2)
Work integration	4 (3.0)
Transition to adulthood	1 (0.7)
Non-applicable (fieldwork study)	1 (0.7)
Not specified	5 (3.7)

^a^ Based on 134 versus 135 studies because Gospodarevskaya et al.'s (2019) study conducted with occupational therapy students. [Gospodarevskaya, E., Carter, R., Imms, C., Yee Chu, E. M., Nicola-Richmond, K. M., Gribble, N., Froude, E., Guinea, S., Sheppard, L., Iezzi, A., & Chen, G. (2019). Economic evaluation of simulated and traditional clinical placements in occupational therapy education. *Australian Occupational Therapy Journal*, *66*(3), 369–379].

^b^ Functional difficulties includes: functional difficulties; residual disability; activity limitations; impaired social functioning; participation restrictions; occupational engagement.

^c^ Activities of daily living difficulties includes: activities of daily living; self-care; bathing disability.

^d^ Job tenure includes: job tenure; absenteeism.

### Occupational Therapy Intervention

The occupational therapy intervention is identified in Table 4. The distinction between the interventions was not always clear. For example, while recognizing the overlap between interventions associated with individuals’ homes (e.g., home-based occupational therapy and fall prevention), the term attributed was usually based on each study's main emphasis (e.g., fall prevention).

**Table 4:** Occupational Therapy Intervention

Occupational therapy intervention evaluated	Frequency (%)
Activities of daily living	54 (40.0)
Rehabilitation	40 (29.6)
Home-based occupational therapy	34 (25.2)
Inpatient rehabilitation	27 (20.0)
Occupation-focused^a^	22 (16.3)
Fall prevention	14 (10.4)
Hospital discharge^b^	11 (8.1)
Home assessment^c^	11 (8.1)
Assistive technology	8 (5.9)
Return-to-work intervention	8 (5.9)
Group intervention	7 (5.2)
Home adaptation	6 (4.4)
Mental health intervention^d^	6 (4.4)
Mobility	6 (4.4)
Therapeutic exercise	5 (3.7)
Lifestyle intervention	5 (3.7)
Cognitive^e^	4 (3.0)
Hospital admission prevention	3 (2.2)
Health problem prevention	3 (2.2)
Mobility assistive device	2 (1.5)
Orthoses	2 (1.5)
Telerehabilitation^f^	2 (1.5)
Transfers	2 (1.5)
Community-based	1 (0.7)
Fieldwork	1 (0.7)
Hand therapy	1 (0.7)
Respiratory rehabilitation	1 (0.7)
Vehicle modifications	1 (0.7)
Vocational rehabilitation^g^	1 (0.7)

^a^ Occupation-focused includes: occupation-focused; activity-based.

^b^ Hospital discharge includes: hospital discharge; early hospital discharge.

^c^ Home assessment includes: home assessment: pre-discharge home visit.

^d^ Mental health intervention includes: mental health intervention; instrumentalism in occupational therapy.

^e^ Cognitive includes: cognitive; cognitive-behavioural.

^f^ Telerehabilitation includes: telerehabilitation; e-health; distance health; online intervention.

^g^ Vocational rehabilitation includes: vocational rehabilitation; job tenure intervention.

The level of detail provided about the occupational therapy intervention varied greatly. For a considerable number of studies, *rehabilitation* or *inpatient rehabilitation* was as precise as could be discerned. The attribution of the term *occupation-based* does not signify that the authors necessarily used the word *occupation*; rather, the nature of the intervention clearly appeared to be occupational in its orientation. Seventy-three (54.1%) studies included more than one type of intervention.

### Research Design, Type of Economic Analysis, and Analysis Perspective

Seventy-two (53.3%) of the studies used a randomized controlled trial. The types of economic evaluation conducted are identified in Table 5; a cost effectiveness analysis was conducted in 63 (46.7%) of the studies.

**Table 5:** Types of Economic Evaluation

Economic analysis	Frequency (%)
Cost effectiveness analysis^a^	63 (46.7)
Cost analysis^b^	35 (25.9)
Cost description^c^	21 (15.6)
Cost utility analysis^d^	14 (10.4)
Cost benefit analysis^e^	10 (7.4)
Cost minimization analysis^f^	4 (3.0)
Social return on investment analysis^g^	2 (1.5)
Analysis of healthcare utilization^h^	1 (0.7)
Cost consequences analysis^i^	1 (0.7)

References: Drummond et al. (2015); Duncan and Larivière (2018).

^a^ Compares the relative costs and outcomes of different interventions, programmes.

^b^ The comparison of costs for the purpose of disclosing and reporting on conditions subject to improvement.

^c^ Breaking down a cost summary into its constituents and studying and reporting on each factor.

^d^ The incremental cost of a programme from a particular point of view is compared to the incremental health improvement expressed in the unit of quality-adjusted life years (QALYs).

^e^ A way to compare the costs and benefits of an intervention, where benefits are expressed in monetary units.

^f^ A method of comparing the costs of alternative interventions (including the costs of managing any consequences of the intervention), which are known, or assumed, to have an equivalent effect.

^g^ A metric used to measure social, environmental, and economic gains that result from an investment.

^h^ The quantification or description of the use of services by persons for the purpose of preventing and curing health problems, promoting maintenance of health and well-being, or obtaining information about one's health status and prognosis.

^i^ This method assesses a wide range of costs and consequences (effects) of the products being compared and reports them separately. It includes all types of effects, including health, non-health, negative, and positive effects, both to patients and other parties (e.g., caregivers).

Although the economic analysis perspective used was not explicitly identified in many of the studies, in most cases, the information provided allowed us to attribute a perspective. Although the costs of the services provided were included in all studies, the spectrum of elements included varied considerably. For example, some analyses only included hospital-based costs (e.g., staff time), whereas others included the costs associated with elements such as staff training, adaptive equipment, home modifications, and transport costs. As well, the distinction was inconsistently made between direct costs (e.g., direct service provider staff time), indirect costs (e.g., hospital readmission) and overheads; given this variability, we have labeled this category *service costs*. How the various kinds of service costs were labeled also varied somewhat depending on who was paying for the services. For example, Björkdahl et al. (2007) reported that the costs were societal since the health and welfare systems in Sweden are tax-financed; however, this was not a societal perspective in the sense of costs, for example, for service users or their caregivers (e.g., productivity loss). Given this variability, our categorization of the economic analysis perspectives included: (a) service costs; (b) service user costs (e.g., out-of-pocket costs, transport, and lost productivity); and (c) caregiver costs (e.g., lost productivity). The authors’ criteria for attributing a societal perspective to their investigations varied considerably; for the purposes of uniformity, we have attributed this term when service user and/or caregiver costs were included in addition to service costs. An explicit or implicit societal perspective was used in 54 (40.0%) investigations. The economic analysis perspective used for each study is specified in the Supplemental Material.

### QHES Score

The average QHES score, based on 133 studies, was 74.4 (out of 100), with 74 (55.6%) investigations obtaining a total score  > 75 (reasonable quality). It was not possible to calculate a score for two studies, in one case because insufficient detail was available in the English version (Toida & Takemura, 2002), and in another case because a qualitative research approach was used (Hutchinson et al., 2020).

### Two Illustrative Cases

Summarizing the overall state of the economic evidence is difficult due to its disparate nature and the limited details provided about the occupational therapy contribution in many investigations. However, two illustrative cases help to lend greater clarity. The home care case is instructive because it is one of the sectors that has received the most attention. In contrast, the case of services for individuals with mental health conditions is useful because the dearth and variability of studies highlight the need for more research. Both areas have been identified as priority needs by the CAOT specialists involved in knowledge transfer and advocacy, and by collaborators with the first two authors in the overall capacity-building project.

#### Case #1: Home Care

The importance of home care services has been well-documented (Canadian Home Care Association et al., 2016). This category includes 32 studies conducted in eight countries. The conditions being experienced by the individuals were specified in the following number of studies: cognitive decline = 8; frailty = 5; arthritis = 1; cerebrovascular accident = 1; chronic illness = 1; diabetes = 1; hypertension = 1; Parkinson's disease = 1; vision challenges = 1. The condition was not specified in 15 studies. The specific nature of the occupational therapy intervention was identified in 27 investigations.

Classifying economic evidence as positive or negative is not always straightforward because this judgment can depend on how much funders are willing to pay to achieve a particular outcome. For example, the intervention for the 75–84 years group in Isarunuwatchai and colleagues’ (2017) Canadian study was cost-effective with a willingness-to-pay to prevent one fall of at least CDN$25,000. Despite this caveat, 13 studies, which included 10 in which the occupational therapy role (sole or interdisciplinary) was specified, more clearly suggested less positive findings. For example, in Sturkenboom and colleagues’ (2015) Netherlands investigation, the home-based occupational therapy intervention for individuals with Parkinson's disease did not significantly impact total costs compared with usual care. In Flood and colleagues’ (2005) United Kingdom (U.K.) study, no significant difference in cost effectiveness was found between occupational therapist-led vs. social worker-led assessment of frail older adults regarding their independence and quality of life.

On the other hand, several studies suggested more positive findings. Five of the seven investigations that included home-based intervention with individuals with cognitive decline and their caregivers, seven of which specified the occupational therapy intervention (sole or interdisciplinary), revealed positive results. For example, in Gitlin and colleagues’ (2010) United States (U.S.) investigation, the *Tailored Activity Program* for individuals with dementia and family caregivers was found to be generally cost-effective for *doing things* and for *being on duty*.

Seven of the 13 studies that specifically targeted fall prevention suggested positive findings, although the details of the specific occupational therapy contribution were not specified in all these investigations. For example, in Xie and colleagues’ (2021) Canadian study, on average, although the *Mobile Integrated Health Care Team* spent approximately 10–15 min longer on scene, the emergency department transport costs were reduced by 45–50%; this team's intervention was associated with savings of approximately 60% of the total cost compared with matched ambulances.

In Zingmark and colleagues’ (2017) Swedish investigation regarding bathing disability being experienced by older adults, the intervention carried out by occupational therapists to minimize this disability resulted in QALY (quality-adjusted life year) gains and reduced societal cost. In Szanton and colleagues’ (2018) U.S. study, the *Community Aging in Place, Advancing Better Living for Elders* programme conducted with individuals with chronic illnesses demonstrated: (a) a 11% lower probability of having an expenditure for any service type; (b) a lower probability of using every service type (except for home health) than the comparison group among people who incurred expenditures; and (c) a significantly lower probability of using inpatient, outpatient, and specialist services, and a higher probability of using home health services.

Some studies provided valuable descriptive information about the costs associated with occupational therapy services. For example, Curtis and Beecham's (2018) U.K. study showed that across major home adaptations, occupational therapists accounted for 18% of staff time; however, across minor adaptations, occupational therapy time accounted for 81% of the total hours.

This brief overview revealed variable results, which suggests that further research is required to understand the reasons for both the positive and less positive findings. As well, given the number of studies in which the details of the occupational therapy contribution were not provided, there is a need for investigations that include these details. The average QHES score (79.3) suggests a reasonable overall rigour of the economic analyses.

#### Case #2: Mental Health Conditions

There is a well-documented unmet need for services for individuals experiencing mental health challenges (Mental Health Commission of Canada, 2021). As well, the need for, and distinct contribution provided by, occupational therapy in responding to these needs has been recognized (American Occupational Therapy Association, 2016). Furthermore, there is a strong economic case to be made for mental health services (Mental Health Commission of Canada, 2017).

Six studies have been conducted in four different countries. The conditions being experienced by the individuals were specified in the following number of studies: psychotic conditions = 3; emotional and behavioural disorders = 1, major depression = 1, and panic disorders = 1. The specific occupational therapy intervention was identified in five investigations.

The three studies (Cook & Howe, 2003; Killaspy et al., 2016; Shimada et al., 2020) regarding occupational therapy interventions for individuals with psychotic conditions demonstrated generally positive results. For example, in Shimada and colleagues’ Japan investigation, adding individual occupational therapy to group occupational therapy (GOT) was associated with a 56.76% probability of being more effective at reducing the rehospitalization rate and a 26.93% probability of being less costly than GOT alone. The need for nuanced interpretation was illustrated by the findings of Cook and Howe's U.K. investigation; although greater costs were associated with providing the intervention, these costs were favorable when compared with similar services.

Positive findings were revealed in both Ikiugu and Anderson's (2007) U.S. study conducted with adolescents with emotional and behavioural disorders, and Schene and colleagues’ (2007) Netherlands investigation conducted with individuals with major depression. On the other hand, in Lambert and colleagues’ (2010) U.K. study conducted with individuals experiencing a panic disorder, no significant difference was found for the group receiving the occupational therapy-led lifestyle approach.

This brief overview suggests that reaching clear conclusions is difficult, beyond the overall limited number of studies, given the range of conditions and interventions across different practice settings (e.g., community; inpatient) and geographic regions. The average QHES score (72.5) suggests a reasonable overall rigour of the economic analyses.

## Discussion

This rapid review of the economic evidence for occupational therapy services has revealed areas of relative strength, some important gaps, and potential directions for future action. From a strength perspective, the average QHES score obtained (74.4) suggests that the overall quality of the economic analyses is reasonable.

Only some of the studies that evaluated the impact of occupational therapy interventions also explored the economic dimension. Therefore, a caveat is that this overview only provides a limited snapshot of the research evidence regarding the impact of certain interventions (e.g., fall prevention). Including both the intervention impact and economic dimension provides more nuanced findings by illustrating positive outcomes (e.g., Gitlin et al., 2010) but also situations in which interventions make a difference for service recipients but are not necessarily economically advantageous (e.g., Sturkenboom et al., 2015).

Our findings suggest that defining occupational therapy's distinct contribution is a necessary ingredient for precisely evaluating the value of the investment. Limited detail about the occupational therapy intervention in approximately 40% of the investigations highlights an important challenge. One possible contributing factor is that many studies were not published in occupational therapy journals. Nevertheless, specifying that occupational therapy is part of the rehabilitation team does not help the profession to make a compelling case. This is not to suggest that occupational therapy should operate solely given the well-recognized importance of interprofessional collaboration (World Health Organization, 2010). Consistent with this perspective, details about interdisciplinary interventions, including occupational therapy, were provided in a considerable number of investigations. Nevertheless, it is essential to identify occupational therapy's contribution, as illustrated by the mental health case example cited earlier, and to measure both its effectiveness for the occupational challenges being experienced as well as the value of the specific resource investment. This approach's importance is highlighted by the value of those studies’ findings in which the occupational therapy role was clearly distinguished. A good example includes Edelstein and colleagues’ (2022) investigation in which individuals with various types of conditions (e.g., cardiovascular problems; pulmonary disease) who received higher frequencies of occupational therapy services while hospitalized had significantly lower odds of readmission. In sum, whether in investigations that solely focus on occupational therapy or those in which it is part of a team intervention, it is essential to provide details about the precise occupational therapy contribution to make the case for its added value.

The systematic reviews that have been published regarding economic evidence for occupational therapy services in specific sectors provide important information. However, beyond these contributions, this rapid review has helped clarify which areas have received attention, but equally importantly, areas that have received little attention. Although some practice sectors (e.g., home care), conditions (e.g., cerebrovascular accident), and age groups (e.g., older adults) have received relatively more attention, this is not the case for many sectors (e.g., mental health), conditions, and age groups (e.g., children and adolescents). It is perhaps more understandable that newer practice approaches such as telerehabilitation have received limited attention to date; the evolution in recent years in this approach to providing services (Abbott-Gaffney et al., 2022) suggests that increased attention is warranted. From the perspective of the individuals experiencing occupational challenges, all sectors, conditions, and age groups merit equal research attention. However, given the relatively young state of economic evidence in occupational therapy, the profession cannot focus on all areas at once. It needs to determine certain priorities that are aligned with clearly identified needs in, for example, government policies and action plans, or strategic plans in health care systems, to maximize the impact of its efforts. In addition to the three areas noted earlier (home care, mental health, telerehabilitation), others might include services for individuals with long COVID (Royal College of Occupational Therapists, 2023), services provided in emergency departments (James et al., 2016), driver evaluation/rehabilitation for older adults (Berndt et al., 2022), and primary care (Donnelly et al., 2023).

The fact that over 40% of the studies were conducted in two countries and over 75% conducted in seven industrialized countries suggests that some prudence is required in the interpretation of their broader applicability. Nevertheless, important challenges regarding the allocation of societal resources exist in many countries (Organisation for Economic Co-operation and Development, 2024). From a strategic perspective, this finding also suggests that the economic evidence studies need to be aligned with the respective demographic trends, resource realities, and organization of services in specific country contexts.

Despite the various gaps that have been noted in the existing economic evidence, the fact that there are several rigorous studies that have been conducted of occupational therapy interventions provides useful models for the profession as it progresses in this area. In a related vein, regarding the types of economic evaluation used, although certain approaches provide more advanced findings, more descriptive approaches (e.g., cost studies) can also be very useful, particularly given the general need for the profession to build capacity in this area. For example, as reported in the home care illustrative case, Curtis and Beecham's (2018) estimate of the total costs associated with supplying and fitting commonly used home adaptations provides highly useful information. Being able to accurately describe the real costs associated with providing occupational therapy services is a logical place for therapists to begin in advancing their economic evidence capacity.

### Strengths and Limitations

To the authors’ knowledge, although some reviews of the economic evidence for occupational therapy services in certain practice sectors have been published, the current review is the first to provide an overview of this literature. This approach has helped to highlight both some global strengths but also some important gaps, which in turn provides the profession with some avenues for strategic advancement.

The decision to only include studies published after 1999 was based on the strategic orientation of a rapid review, that is, maximizing its relevance given the significant evolution that has occurred in occupational therapy practice in recent decades. However, this decision may constitute a limit on this overview's comprehensiveness. Second, there may be economic analyses published in languages other than English and French. Third, we noted earlier the concentration of research in a limited number of countries. Finally, the fact that limited information was provided about the specific occupational therapy contribution in many studies means that some prudence is required regarding the interpretation of this contribution.

### Implications for Occupational Therapy Practice

Occupational therapy practitioners, in collaboration with other profession stakeholders (e.g., professional associations), have an important role to play in defining their distinct contribution. This information is an essential ingredient for developing economic analyses of occupational therapy services, including within studies that measure the costs associated with services provided by multiprofessional teams. Practitioners can work in partnership with researchers conducting economic analyses given their specific knowledge and experience about their practice (Duncan et al., 2018). Practitioners can make an important contribution by measuring the costs of their services; beyond the intrinsic value of these cost descriptions, they are an important first step towards conducting more sophisticated economic analyses.

## Conclusion

This rapid review of the economic evidence for occupational therapy services reveals that although some practice sectors, conditions, and age groups have received attention, considerable gaps exist. Furthermore, in many investigations, the precise contribution of occupational therapy has not been specified. These findings suggest that the profession should identify strategic priorities for advancing the economic evidence and ensure that research includes precision about occupational therapy's added value.

## Key Messages

- The economic evidence for occupational therapy services is highly variable, concentrated in a limited number of jurisdictions, and with important gaps in certain areas (e.g., mental health and children).
- The overall evidence is hampered by the frequent absence of details about the occupational therapy contribution.
- This rapid review helps to identify strategic approaches for advancing the profession's economic evidence capacity.

## Supplemental Material

sj-docx-1-cjo-10.1177_00084174241306983 - Supplemental material for Economic Evidence in Occupational Therapy: A Rapid Review Supplemental material, sj-docx-1-cjo-10.1177_00084174241306983 for Economic Evidence in Occupational Therapy: A Rapid Review by Andrew R. Freeman, Nadine Larivière, Judith Baillet, Rachel Beauchemin, Étienne Lavoie-Trudeau, Myriam Martel and Mégan St-François in Canadian Journal of Occupational Therapy
