# Supplementary material for: Economic Evidence in Occupational Therapy: A Rapid Review
Source: Can J Occup Ther. 2025 Jan 6;92(3):173–84. doi: 10.1177/00084174241306983 (PMC12326029; doi:10.1177/00084174241306983)
Supplement: sj-docx-1-cjo-10.1177_00084174241306983 - Supplemental material for Economic Evidence in Occupational Therapy: A Rapid Review [file sj-docx-1-cjo-10.1177_00084174241306983.docx]

**Supplementary Information: Economic evidence in occupational therapy: A rapid review**

**Table 1: Article Summaries**

| **Authors**  **Year**  **Country** | **Objective** | **Age Group & Condition** | **OT* intervention**  **evaluated** | **Clear OT role**  ****** | **Research design, economic analysis approach & perspective** | **Economic Outcomes** | **QHES Score** |
| --- | --- | --- | --- | --- | --- | --- | --- |
| Ah-Soune & De Vignerte (2000)  France | To evaluate the effectiveness of the reintegration process (Follow-up Care & Rehabilitation - SSR), in particular, through the occupational therapy intervention, as well as the reintegration cost for the payers, in order to identify an acceptable cost-effectiveness ratio for the different stakeholders. | - older adult - orthopedic | - home assessment - hospital discharge | No | - prospective before-and-after - cost analysis - cost effectiveness analysis - Perspective: service costs, service user costs | The cost was greater for the Follow-up Care & Rehabilitation (SSR) and for the participants in the Home Visit group. However, the relative cost for effective maintenance at home after 2 months was minimal for the SSR and the patient, and nil for the community. | 70 |
| Allen et al. (2019)  Canada | To evaluate the cost-effectiveness of the Community Stroke Rehabilitation Team (CSRT) programme compared with a “usual care” cohort. | - adult/older adult - stroke | - home-based occupational therapy | No | - pre-test/post-test - cost effectiveness analysis - Perspective: service costs | The Community Stroke Rehabilitation Team (CSRT) programme demonstrated a net monetary benefit of CDN$43,655 over Usual Care, and was both less costly and more effective. The incremental cost-effectiveness ratio of the CSRT programme is superior in 100% of iterations when compared to Usual Care. | 78 |
| Anderson et al. (2000)  Australia | To examine the resource and economic implications of an early hospital discharge and home-based rehabilitation scheme for patients with acute stroke. | - older adult - stroke | - hospital discharge - home-based occupational therapy | No | - randomized controlled trial - cost minimization analysis - Perspective: societal (service costs, caregiver costs) | Although the mean cost/patient was lower for patients randomized to the early hospital discharge and home-based rehabilitation compared with those who received conventional care, this cost saving was not statistically significant. Only when the costs of care in hospital were assumed to be 50% of baseline did the conventional care scheme become cheaper. The cost of the home-based program was significantly related to a patient’s level of disability after adjustment for age, comorbidity, and the presence or absence of a caregiver. | 86 |
| Andersson et al. (2002)  Sweden | To analyse whether a redistribution of costs occurs between health care providers and social welfare providers in a comparison of home-based and hospital-based rehabilitation after stroke. | - older adult - stroke | - home-based occupational therapy - inpatient rehabilitation | No | - prospective observational cohort study - cost analysis - Perspective:service costs | The total costs for the care episode did not differ between the two groups. The hospital-based rehabilitation group had  significantly fewer hospitalization days after a decision was made about rehabilitation at the acute care ward; consequently, the cost for the acute care period was significantly lower. The cost for the rehabilitation period was  significantly lower in the home-based rehabilitation group; however, the cost for home help service was significantly higher in this group. | 33 |
| Angelo et al. (2021)  United States | To explore the association between therapy and total cost of care for the frail elderly population. | - older adult - frailty | rehabilitation | No | - retrospective comparative study - cost description - Perspective: service costs | When the frail population was segmented into those who receive outpatient rehabilitation therapy and those who did not, outpatient rehabilitation therapy is associated with decreased total care costs at 13–32 therapy units delivered. However, outside of this range of therapy units, outpatient rehabilitation therapy was not associated with statistically significant reductions in total cost of care. | 100 |
| Angerová et al.  (2021; 2020)  Czech Republic | (1) To calculate the hospitalization costs in patients after stroke at stroke units during the early rehabilitation therapy. (2) To determine whether the cost and cost-effectiveness of early rehabilitation are associated with the degree of initial disability. | - adult/older adult - stroke | inpatient rehabilitation | No | - prospective observational cohort study - cost analysis - cost effectiveness analysis - Perspective: service costs | Although there were significant differences in 1-day hospitalization costs between hospitals, the structure of direct costs was quite stable. Costs grew with the degree of disability; depending on the disability category, the cost increase was between 56-143%. The main driver of the increase was nursing cost. No more than 15% of total costs were attributed to rehabilitation therapists. | 85 |
| Archongka et al. (2008)  Thailand | To determine whether the current allowance of 150 Baht per visit is sufficient for the rehabilitation of stroke patients. | - adult/older adult - stroke | inpatient rehabilitation | No | - prospective before-and-after - cost analysis - Perspective: service costs | Although the total rehabilitation cost at Sirindhron National Medical Rehabilitation Center was higher than those at the multi-center, the difference was not statistically significant. | 26 |
| Bendixen et al. (2009)  United States | To investigate the health-related cost analyses between the Veteran Health Administration’s Care Coordination Home Telehealth telerehabilitation program [Low Activities of Daily Living Monitoring Program (LAMP)] and a matched comparison group (MCG) receiving VHA standard care. | - adult/older adult - arthritis - cardio-vascular problem - chronic illness - stroke | - activities of daily living - assistive technology - home adaptation - telehealth | No | - quasi-experimental - cost analysis - Perspective: service costs | Total costs for hospital bed days of care decreased 46% in the year following enrollment. No significant differences were detected in post-enrollment costs between Low Activities of Daily Living Monitoring Program (LAMP) and the matched comparison group. For LAMP patients, the provision of adaptive equipment and environmental modifications, plus intensive in-home monitoring of patients, led to increases in clinic visits post-intervention with decreases in hospital and nursing home stays. | 48 |
| Björkdahl et Sunnerhagen (2007)  Sweden | To describe the direct and indirect costs of hospitalization and rehabilitation in the first year after a stroke in "younger" persons (<65 years) and to examine the factors that contribute to higher costs. | - adult - stroke | - home-based occupational therapy - inpatient rehabilitation - home adaptation | No | - randomized controlled trial - cost description - cost effectiveness analysis - Perspective: service costs | Inpatient care contributed substantially to the direct cost with a mean length of stay of 92 days. Rehabilitation during the first year constituted of an average of 28 days in day clinics, 38 physiotherapy sessions and 20 OT sessions. The direct costs were influenced by the process skills (the ability to plan and perform a given task and to adapt when needed) and presence of aphasia. Indirect costs for informal caregiving increased for patients with a lower health-related quality of life as well as a low score on home integration. | 51 |
| Breysse et al. (2022)  United States | To conduct a randomized controlled effectiveness study of the CAPABLE (Community Aging in Place-Advancing Better Living for Elders) program’s impact on ADL and IADL limitations and other function-related parameters. | - older adult - not specified | - fall prevention - home-based occupational therapy | No | - randomized controlled trial - cost analysis - Perspective: service costs | The median costs for the 4 locations were highly variable. The intervention group showed a slight decrease in the percentage of participants who went to the Emergency Room (ER) and were subsequently hospitalized for at least one night, whereas the control group showed a slight increase; however, these changes were not significant. The % of participants who visited the ER at least once (but were not hospitalized overnight) increased slightly for the intervention group but decreased for the control group. Both groups showed reductions in mean unplanned health care cost rates between 1 year pre-baseline and 1-year post-baseline. The total 1-year post-baseline mean cost rate for the intervention group was slightly less than that for the control group; however, the control group’s cost reduction exceeded that of the intervention group. | 76 |
| Brusco et al. (2014)  Australia | To determine from a health service perspective if the provision of a rehabilitation service to inpatients on a Saturday in addition to Monday to Friday compared to Monday to Friday rehabilitation alone, was cost effective per quality adjusted life year (QALY) gained and for a minimal clinically important difference (MCID) in functional independence. | - adult - mixed neurological conditions - orthopedic - stroke | inpatient rehabilitation | No | - randomized controlled trial - cost effectiveness analysis - Perspective: service costs (including unplanned readmissions) | The incremental cost utility ratio showed a cost saving of AUD$41,825 per QALY gained for the intervention group compared to the control group. The incremental cost effectiveness ratio showed a cost saving of AUD$727 for a 1-point change in the functional independence score for the intervention group compared to the control group. Patients in the intervention group were 17% more likely to achieve a minimally clinically important difference in functional independence at discharge compared to those in the control group. | 93 |
| Brusco et al. (2015)  Australia | To determine from a health system perspective inclusive of private costs if the likely short-term cost effectiveness of providing an additional Saturday rehabilitation service to inpatients in addition to Monday to Friday compared to Monday to Friday rehabilitation alone, is sustained 12 months following discharge from rehabilitation. | - adult/older adult - mixed neurological conditions - orthopedic | inpatient rehabilitation | No | - randomized controlled trial - cost effectiveness analysis - Perspective: societal (service costs, service user out-of-pocket costs) | From admission to 12 months, there was a non-significant reduction in cost. However, from admission to 6 months, there was a significant reduction in cost. Sensitivity analyses varying the cost of informal carers and self-reported health service utilization favored the intervention. | 79 |
| Brusco et al. (2022)  Australia | Within the context of a capabilities approach (CA), this study aimed to investigate the economic viability of robotics-based therapy used in upper limb stroke rehabilitation in rural Victoria, Australia. | - adult/older adult - stroke | - inpatient rehabilitation; outpatient rehabilitation - rehabilitation centre | No | - quasi-experimental - cost analysis - Perspective : societal (service costs, service user out-of-pocket costs & transport, caregiver productivity losses) | The Functional Independence Measure (FIM) demonstrated observed improvements in the functional score for both cohorts from baseline to post-intervention, yet there were no within-group, between-group or subgroup scores that achieved or trended towards statistical significance (70-80% data missing for the usual care group). The quality of life (EQ5D3L) demonstrated no differences in the robotics cohort (100% missing data for the usual care group). While there was an observed reduction in acute admissions and outpatient session utilisation and cost for the robotics cohort, there was a significant reduction in rehabilitation admissions and cost for the usual care cohort. The health service cost of usual care 1:1 out-patient rehabilitation was consistently higher, indicating that an established RBT program may be cost-effective, specifically providing less cost for the same effect. | 85 |
| Burns et al. (2016)  United Kingdom | To conduct an economic evaluation alongside a RCT of a low-intensity (2 hour) maintenance programme for individuals with COPD over a time horizon of 1 year delivered in UK primary and secondary care settings. | - adult/older adult - pulmonary disease | - group intervention - health problem prevention | No | - randomized controlled trial - cost effectiveness analysis - Perspective: societal (service costs; service user out-of-pocket costs & productivity losses) | The intervention was less expensive, but yielded worse outcomes on one measurement scale (Chronic Respiratory Questionnaire) but better outcomes on another (QALY). The intervention is approximately equally likely to be cost saving or cost incurring. There was a 72.88 % probability of cost effectiveness at a threshold of £20,000 per QALY, declining slightly to 72.52 % at a £30,000 threshold. | 69 |
| Campbell et al. (2005)  New Zealand | To investigate the effectiveness of 2 home-based strategies to prevent falls in elderly people with impaired vision – a programme to address safety in the home environment and a programme of strength and balance retraining plus vitamin D supplements. | - older adult - significant vision challenges | - fall prevention - home assessment | No | - randomized controlled trial - cost effectiveness analysis - Perspective: societal | As it was not found that the exercise programme was effective in reducing falls (although stricter adherence was associated with fewer falls), only the cost effectiveness of the home safety programme was evaluated. The programme cost NZ$64 337 ($325/person). The incremental cost per fall prevented was NZ$460; estimates ranged from NZ$460-1569/fall prevented for the different cost scenarios. | 92 |
| Carande-Kulis et al. (2015)  United States | To identify fall interventions that were feasible, effective, and provided a good return on investment (ROI). | - older adult - frailty | fall prevention | Yes | - retrospective observational design - cost benefit analysis - social return on investment analysis - Perspective: service costs | All three interventions were cost-saving. They demonstrated positive net benefits, that is, the benefits from averted direct medical costs outweighed the costs of implementing the intervention. The Tai Chi and Otago Exercise Program delivered to persons aged 80 and older yielded returns on investment (ROIs) greater than 100% (substantial returns). Tai Chi had the highest ROI; The average intervention cost was highly influenced by both personnel salary costs and the intervention format (group versus individual, frequency per week). | 44 |
| Carlill et al. (2002)  United Kingdom | To investigate whether an occupational therapy and social work service was needed in the NHS trust’s Accident and Emergency (A&E) Department to facilitate safe discharge and to prevent unnecessary admissions. | - older adult - not specified | - hospital discharge - hospital admission prevention | No | - retrospective chart review - cost analysis - Perspective: service costs | The service was beneficial with respect to both preventing unnecessary hospital admissions and arranging suitable community support when patients were discharged home from the Accident & Emergency department. A potential 500 bed days were saved because of the service. | 29 |
| Chen et al. (2022)  Taiwan | To measure functional outcomes and conduct cost-utility analysis of an organized multidisciplinary postacute care (PAC) project in secondary care compared with standard rehabilitative care delivered in tertiary care. | - adult/older adult - stroke | - activities of daily living - cognitive - inpatient rehabilitation - mobility - occupation-focused - rehabilitation |  | - prospective observational cohort study - cost utility analysis - Perspective : service costs | Cost-utility analysis revealed that the PAC group had a significantly lower mean of direct medical costs and a significantly higher average gain of quality-adjusted life years. At a willingness-to-pay threshold of US$26,263.5 per QALY, the PAC project had a 100% likelihood of being cost effective compared to standard rehabilitation. | 97 |
| Chew et al. (2022)  United States | To report the economic outcomes of the REHAB-HF trial and estimate the potential cost-effectiveness of the intervention. | - older adult - cardio-vascular problems | rehabilitation | No | - randomized controlled trial - cost effectiveness analysis - Perspective: service costs | The mean medical costs were similar in both groups, but quality-of-life gains were greater in the rehabilitation intervention group. Lifetime cost-effectiveness ratios for the intervention varied, but most were within conventional benchmarks for good value when simulated using the validated *Tools for Economic Analysis of Patient Management Interventions in Heart Failure Cost-Effectiveness Model*. | 97 |
| Chou et al. (2023)  Taiwan | To explore the changes in functional ability, walking ability,  and national health insurance (NHI) cost for different hospitalization durations and the effects of functional recovery and regaining walking ability on NHI costs. | - adult/older adult - stroke | - activities of daily living - rehabilitation | No | - retrospective observational design - cost effectiveness analysis - Perspective: service costs | The changes in national health insurance (NHI) costs varied depending on whether hospitalization was extended. At any time point, functional performance did not have a significant impact on NHI cost. Age (higher the age, the lower the NHI cost at week 3) and sex (NHI cost was higher in male patients) had significant effects on NHI cost. | 45 |
| Clare et al. (2023)  United Kingdom | The aim of this translational study, building on evidence from the GREAT randomised controlled trial, was to develop a foundation for implementing the GREAT Cognitive Rehabilitation intervention in community-based services for people with mild-to-moderate dementia. | - adult/older adult - cognitive decline | cognitive | No | - quasi-experimental - cost analysis - Perspective: service costs | Goal attainment by people with dementia exceeded levels of improvement seen in the original trial. The intervention could be provided at modest cost. Excluding travel time, the total cost of 6 sessions provided by a qualified OT was £349, which reduced to £239 if the first and last sessions were provided jointly with an OT assistant who conducted the intervening 4 sessions. A range of mainly organisational barriers that impeded implementation and limited the potential for sustainability were identified. | 57.6 |
| Clark et al. (2012)  United States | To determine the effectiveness and cost-effectiveness of a preventive lifestyle-based occupational therapy intervention, administered in a variety of community-based sites, in improving mental and physical well-being and cognitive functioning in ethnically diverse older people. | - older adult - cognitive decline | lifestyle intervention | Yes | - randomised controlled trial - cost effectiveness analysis - Perspective: service costs | The intervention group had a significantly greater increment in quality-adjusted life years, which was achieved cost-effectively. The estimated base case cost per QALY of US$41 218/ UK£24 868 is low enough for the intervention to qualify as cost-effective. | 70 |
| Clarke et al. (2016)  United Kingdom | To evaluate the clinical effectiveness and cost-effectiveness of individualised physiotherapy and occupational therapy in patients with Parkinson’s Disease. | - adult/older adult - Parkinson’s disease | - assistive technology - home adaptation - home-based occupational therapy | No | - randomized controlled trial - cost effectiveness analysis - Perspective: service costs | Although PT/OT* was associated with a small gain in QALYs at a small incremental cost, the difference was not statistically significant. The incremental cost per QALY was under £4000 but highly uncertain. At a willingness-to-pay threshold of £20,000 per QALY, the probability of PT/OT being more cost-effective at £20,000 was 50.5%. | 94 |
| Cook & Howe (2003)  United Kingdom | To investigate the feasibility of a primary care based service for people with psychotic conditions who were not in contact with a secondary care based generic community mental health teams. To find out if the direct costs of the primary care based mental health service were comparable with those of similar community services. | - adult - mental health condition | mental health intervention | Yes | - mixed methods - quasi-experimental - pre-post test design - case study - cost benefit analysis - Perspective: service costs | After 12 months intervention, (a) the participants’ mean overall score for social functioning improved; (b) the % of the participants that had a clinical level (psychiatric symptoms) was reduced in 10 of the 12 symptom areas; (c) the participants’ mean scores for most of the problem areas measured by the *Health of the Nation Outcome Scales* were reduced. The total cost per person per year of direct NHS mental health care services was estimated as £183 before the start of the new service and £1767 after the new service started, showing an increase in cost of £1584 for the new service. The increase in cost for community and outpatient care was estimated as £813 and for hospital admissions as £771. These costs were favorable when compared with similar services. | 75 |
| Cooney & Carroll (2016)  Ireland | To examine the reduction in direct care costs after acquired brain injury that can be achieved through specialist rehabilitation at the tertiary specialist rehabilitation centre by assessing the changes in functional ability of patients with brain injury after a period of inpatient rehabilitation and the financial savings in terms of cost of care accrued as a result of these improvements in functionality. | - adult - brain injury - stroke | inpatient rehabilitation | No | - prospective before-and-after - cost effectiveness analysis - Perspective: societal (service costs, service user contribution to residential costs) | The average care costs on admission of the group were €629.10 weekly, which fell to €242.20 weekly at discharge (reduction of €386.90). The average length of time for the cost of inpatient stay to be covered by the savings in terms of reduced care costs was 30 months. The average care costs on admission of the group were €629.1 weekly, which fell to €242.2 weekly at discharge. At a daily inpatient cost of €700, the average cost of inpatient rehabilitation was €48,949 per person. The average length of time for the cost of inpatient stay to be covered by the savings in terms of reduced care costs was 30 months. | 56 |
| Cunningham et al. (2009)  United States | To examine the direct cost savings associated with a reduction in medication use by patients with chronic nonmalignant pain following the completion of a 3-week outpatient pain rehabilitation program and to determine if cost savings were sustained. | - adult/older adult - pain | - cognitive - occupation-focused | No | - pre-post study - cost analysis - Perspective: service costs (medication) | Statistically significant medication cost savings were seen for program completers at discharge and at 6-month follow-up. The mean daily prescription medication cost savings from admission to 6-month follow-up was US$6.68. | 60 |
| Curtis & Beecham (2018)  United Kingdom | To estimate the total costs of supplying and fitting commonly used home adaptations. | - adult/older adult - not specified | home adaptation | No | - description - cost description - Perspective: service costs | Across all major adaptations, OTs accounted for 18% of staff time. The lowest time inputs were required for straight stairlifts (OT time 7%), although this accounts for a relatively high proportion of total costs (24%). The highest staff costs were for downstairs bedroom/bathroom extensions absorbing 80 staff hours (OT time = 22%). Considerable variations in staff time were observed for each adaptation. Increased staff time was reported as being due to higher user needs. Across all minor adaptations, OT time accounted for 81% of the total hours. For 40% of the items, an OT assistant or support worker either carried out the assessment or was also present. On average for minor adaptations, OT costs absorbed 51% of total staffing costs, although there was considerable variable across types of adaptations. | 76 |
| Duru et al. (2009)  United States | To calculate intervention costs and the potential cost offset of a care management intervention that substantially improved the quality of dementia care. | - older adult - cognitive decline | home-based occupational therapy | No | - randomized controlled trial - cost analysis - Perspective: societal (service costs, caregiver opportunity costs) | The intervention required a start-up cost of US$70,256 and mean intervention per-patient per-month costs of US$118. There were no significant differences in the mean monthly cost of healthcare and caregiving services for intervention versus usual care patients using the societal perspective or the payer perspective. | 85 |
| Edelstein et al. (2022)  United States | To identify the impact of occupational therapy services receipt, duration, and frequency on readmissions and to compare the types of occupational therapy services delivered to patients who were readmitted versus not readmitted on the basis of Current Procedural Terminology codes. | - older adult - cardiovascular problems - pulmonary disease - orthopedic | - inpatient rehabilitation - hospital discharge - hospital admission prevention | Yes | - retrospective chart review - analysis of healthcare utilization - Perspective: service costs | Patients who received OT services while hospitalized had significantly higher odds of readmission. Patients who received higher frequencies of OT services had significantly lower odds of readmission. Patients who received higher duration of OT services did not have significantly higher or lower odds of readmission. A higher proportion of not-readmitted patients received ADL/self-care training. | 89 |
| Eklund et al. (2021)  Sweden | To evaluate the cost-effectiveness of Multimodal Rehabilitation Programs (MMRPs) at one-year follow-up in comparison with care as usual for patients with chronic pain in primary healthcare in two Swedish regions. | - adult - pain | rehabilitation | No | - prospective observational cohort design - cost utility analysis - Perspective: societal (service costs, service user productivity losses) | The Multimodal Rehabilitation Programs (MMRPs) were cost-effective compared with care as usual when implementing a common cost effectiveness threshold of 19,734 €. The MMRP participants’ health-related quality of life increased significantly. The number of patients not on sickness absence increased by 15% and contributed to cost savings in terms of reduced loss of production. | 92 |
| Ellis et al. (2006)  United Kingdom | To determine the NHS and Social Services resource use and cost-effectiveness of a form of intermediate care (stay in a residential rehabilitation unit) as compared with ‘usual’ community services at home, for older people on discharge from hospital. To estimate the average costs of these two models of service provision as a guide to policy decision-makers considering the provision of such units in other parts of the country. | - adult/older adult - not specified | - activities of daily living - inpatient rehabilitation - occupation-focused - mobility | No | - retrospective chart review - cost effectiveness analysis - Perspective: service costs | Overall, combined NHS and Social Services costs in the two groups were similar. However, there was a clear « seesaw » effect between the NHS and Social Services: the cost of the unit option fell more heavily on Social Services, the community option more so on the NHS. This result suggests that residential rehabilitation for older people is no more cost-effective over a year after discharge from community hospital than usual community services. | 77 |
| Evans et al. (2016)  United States | To examine the cost-effectiveness of one interdisciplinary pediatric chronic pain rehabilitation program by comparing the economic impact of pain at admission and one year later. | - Adolescent/adult - pain | - inpatient rehabilitation - rehabilitation | No | - retrospective chart review - cost effectiveness analysis - Perspective: societal (service costs, caregiver productivity losses) | For the participants in the rehabilitation program, there were statistically significant reductions in days hospitalized, physician office visits, physical/OT services, psychotherapy visits, and parental missed work. The difference in total estimated costs in the year before  admission and total estimated costs in  the year after admission minus the cost of the program itself translated to an estimated savings of US$27,119 per family in the year following admission. | 72 |
| Everink et al. (2018)  Netherlands | To determine the cost-effectiveness and cost-utility of an integrated care pathway designed for community-dwelling older adults transitioning from the hospital to a post-acute geriatric rehabilitation facility, and then to home. | - older adult - frailty - not specified | rehabilitation | No | - prospective observational cohort study - cost effectiveness analysis - cost utility analysis - Perspective: societal perspective (service costs, service user & caregiver costs) | After 9 months, the average societal costs were significantly lower for patients in the care pathway cohort.  Patient and family costs did not significantly differ between the cohorts. The probability of the integrated care pathway being cost-effective when compared to care as usual remains 99% or higher for a range of willingness-to-pays. The probability of the integrated care pathway being cost-effective, compared to care as usual, is 98%. No differences in QALY were detected. | 100 |
| Firzah Abdul Aziz et al. (2020)  Malaysia | To undertake a cost effectiveness analysis of the implementation of the integrated care pathway for managing post stroke patients (iCaPPS©) compared to conventional post stroke care from societal perspective. | - adult/older adult - stroke | rehabilitation | No | - randomized controlled trial - cost effectiveness analysis - Perspective : societal (service costs, service users, caregivers) | The cost for post-stroke monitoring with iCaPPS was almost 50% higher than the costs of conventional care. This difference is due to the increased provider costs, which were mainly driven by the staff salaries and drugs. However, the outcome of iCaPPS regarding costs per QALY is 12.7% lower than conventional care. | 93 |
| Flood et al. (2005)  United Kingdom | To compare costs and outcome of occupational therapy-led assessment with social worker-led assessment of older people, in terms of their independence and quality of life. | - older adult - frailty | home assessment | Yes | - randomized controlled trial - cost effectiveness analysis - Perspective: societal (service costs, service user & caregiver out-of-pocket costs) | The mean primary and community care costs were equivalent for both arms. There were no differences between the arms of the trial in terms of cost-effectiveness. Although there is an increase in mean cost/case for the OT arm, it is not significant. The analysis based on QALYs generated by combining life expectancy data and quality of life data was the only case where the probability of a marginal cost-effectiveness difference reached 50%. At best this means having to spend sums of up to £14,000 for a QALY gain, but in 50% of simulated analyses the OT intervention was not relatively cost-effective at any threshold. | 79 |
| Gage et al. (2006)  United Kingdom | To investigate, by means of a case study, how cost-consequences analysis can be used as an evaluative tool, and to discuss some practical limitations. | - adult/older adult - Parkinson’s disease | - group intervention - rehabilitation | No | - case study - cost consequences analysis - Perspective : societal (service costs, service user transport costs) | The largest elements of cost were associated with the use of facilities (treatment rooms and communal space) in the day hospital, staff time, and transport for people unable to provide their own. | 56 |
| Gieser et al. (2006)  United States | To examine the total resource utilization and resulting costs related to end-stage glaucoma through a retrospective chart review. To assess real-world ophthalmic medical management and use of Vision Rehabilitation Care (VRC) services in patients with end-stage glaucoma including direct costs of glaucoma management and VRC. | - adult/older adult - significant vision challenges | - activities of daily living - assistive technology | No | - retrospective chart review - cost description - Perspective: service costs | The mean number of visits was 7.1 in year 1 and 3.7 in year 2, for an annual mean of 5.4 visits overall. Of the total 2-year costs, 15% were Vision Rehabilitation Care (VRC) costs, 37% were ophthalmology costs, and 48% were pharmacy costs. Further analysis of the nonpharmacy costs revealed that VRC costs accounted for 28%, and ophthalmology costs for 72%, of the 2-year medical costs. Overall charges were slightly higher for patients under age 65 versus those aged 65 and older. | 50 |
| Gillespie et al. (2022)  Ireland | To examine the cost-effectiveness of a 6-week occupational therapy-led self-management support programme (OPTIMAL) for adults with multimorbidity. | - adult/older adult - multimorbidity | - health promotion - lifestyle intervention - occupation-focused | Yes | - randomized controlled trial - cost effectiveness analysis - Perspective : service costs | A mean improvement in QALYs gained of 0.031 per patient (P-value: 0.063; 95% confidence intervals [CIs]: −0.002 to 0.063) and a mean reduction in total costs of €2,548 (P-value: 0.114; 95% CIs: −5,606 to 509) per patient. At cost-effect¬iveness threshold values of €20,000 and €45,000 per QALY, the probability of the intervention being cost-effective was estimated to be 0.951 and 0.958, respectively. The results remained consistent across all subgroups examined. | 100 |
| Gitlin et al. (2010)  United States | To evaluate cost-effectiveness of the Tailored Activity Program (TAP) for individuals with dementia and family caregivers. | - older adult - cognitive decline | - occupation-focused - home-based occupational therapy | Yes | - randomized controlled trial - cost effectiveness analysis - Perspective: societal (service costs, caregiver costs) | The incremental cost-effectiveness ratio showed that intervention caregivers saved one extra hour per day “doing things” at a cost of US$2.37 per day; and one extra hour per day “being on duty” at a cost of US$1.10 per day. The Tailored Activity Program (TAP) was cost-effective 79.2% of the time for “doing things” and 79.6% of the time for “being on duty.” | 87 |
| Godwin et al. (2011)  United States | To accurately cost account comprehensive, outpatient rehabilitative services and medications for stroke survivors and to describe trends in utilization over the first year after discharge from inpatient rehabilitation in a sample of first-time stroke survivors from 5 hospitals in Southeast Texas. | - adult/older adult - stroke | - home-based occupational therapy - inpatient rehabilitation | No | - pre-test/post-test - cost analysis - Perspective: service costs (including specific focus on medication costs) | Cost attributed to medication remained relatively constant throughout the 3 groups (dependent, modified dependence, independent). Outpatient rehabilitation service utilization constituted a large portion of cost within each group: 69.7% (dependent),  72.5% (modified dependence), and 66.7% (independent). Apart from those who had severe functional limitations at discharge home, the total average cost for service utilization was highest during the first 3 months and decreased throughout the year, while the average cost of medication remained relatively constant over the course of the year for all 3 groups. | 53 |
| Gospodarevskaya et al. (2019)  Australia | To present detailed cost analyses of two placement alternatives and a cost-benefit study to assess the value for money of simulated clinical placements. | - OT student - fieldwork | fieldwork | Yes | - randomized controlled trial - cost analysis - cost benefit analysis - Perspective: service costs (associated with providing placements) | From the universities’ perspective the average cost per student ranged from AUD$460-1511 for simulated (SCP) and AUD$144-1112 for traditional (TCP) placement. From the health care sector perspective, the difference in costs favoured simulated placements for four implementations and traditional placements for five. In the Discrete Choice Experiment, respondents preferred traditional rather than  simulated placement and would pay additional AUD$533. The estimated monetary value of simulated placements from a contingent valuation ranged from AUD$200-1600. | 86 |
| Graff et al. (2008)  Netherlands | To assess the cost effectiveness of community based occupational therapy compared with usual care in older patients with dementia and their care givers from a societal viewpoint. | - older adult - cognitive decline | community-based | Yes | - randomized controlled trial - cost effectiveness analysis - Perspective: societal (service costs, caregiver productivity losses) | Visits to general practitioners and hospital doctors cost the same in both groups; however, total mean costs were €1748 lower in the intervention group, with the main cost savings in informal care. The acceptability curve shows that if society is willing to pay €2000 or more for a successful treatment, then there is 99% probability that OT is efficient. | 86 |
| Griffiths et al. (2001)  United Kingdom | Assess the costs and benefits resulting from a 6-week outpatient rehabilitation programme which was being evaluated in a randomized controlled study of its clinical effectiveness following its introduction in an NHS hospital in South Wales (UK). | - older adult - pulmonary disease | respiratory rehabilitation | No | - randomized controlled trial - cost utility analysis - Perspective: societal (service costs, service user transport costs) | No significant difference was observed between the control and rehabilitation groups in relation to the overall cost of their care. The programme resulted in an increase in the mean number of QALYs generated of 0.03 per patient and a non-significant mean cost saving of £152 per patient. The cost per QALY of providing rehabilitation has a probability of 0.7 of being within an acceptable range. | 96 |
| Grimmer et al. (2001)  Australia | To explore the impact of potential cost drivers of ambulatory occupational therapy episodes of care. | - children/adolescent/adult/older adult - not specified | rehabilitation | Yes | - retrospective observational design - cost analysis - Perspective: service costs | The median value of the total patient-attributable time of an OT episode of care was 70 minutes. The factors that exerted a significant influence on the length of the completed OT episode of care were age, communication difficulties and hospital location. If patients were aged 5 years or less, they had a significantly increased likelihood of requiring longer episodes of care compared with patients aged 75 years or more. Patients aged 75 years or more had significantly increased likelihood of requiring longer episodes of care than patients aged between 17 and 74. Patients judged by OTs to have communication difficulties were significantly more likely to have longer episodes of care than patients without such difficulties. Patients treated in a metropolitan hospital had longer episodes of care than patients treated in rural settings. Regarding the findings from the univariate logistic regression models, there were no noteworthy non-significant results, nor were there identifiable trends. | 38 |
| Haines et al. (2017)  Australia | To establish the impact of disinvesting from provision of allied health services on weekends across acute medical and surgical hospital wards. | - adult/older adult - orthopedic - stroke - pulmonary disease | rehabilitation | No | - randomized controlled trial - cost analysis - Perspective: service costs | In Trial 1, criteria to say that the no weekend allied health condition was non-inferior to current weekend allied health condition were not met, while neither the no weekend nor current  weekend allied health condition demonstrated superiority. In Trial 2, the no weekend allied health condition was non-inferior to the newly developed weekend allied health condition across all primary outcomes, and superior for the outcomes proportion of patients staying longer than expected, proportion experiencing any adverse event, and mean length of stay. | 92 |
| Harper et al. (2024)  Australia | To evaluate an occupational therapy delirium pathway facilitating early assessment, intervention and supported discharge to home compared with hospital-based care. | - older adult - delirium | - activities of daily living - cognitive - early hospital discharge - fall prevention - home adaptation - home assessment - home-based occupational therapy | Yes | - randomized controlled trial - cost analysis - Perspective: service costs | No significant difference in (a) the length of stay in hospital or (b) the inpatient occupational therapy time with patients. The control group had significantly more hospital re-presentations. No significant factors were associated with hospital re-presentations. |  |
| Hay et al. (2002)  United States | To evaluate the cost-effectiveness of a 9-month preventive occupational therapy program in the Well-Elderly Study (Lifestyle Redesign). | - older adult - not specified | lifestyle intervention | Yes | - randomized controlled trial - cost effectiveness analysis - Perspective: societal (service costs, caregiver costs) | Although no significant differences were detected in healthcare and caregiver costs across the treatment groups during the treatment phase or the posttreatment phase, healthcare costs at follow-up were substantially lower for the OT group than for the active control group, the passive control group, or the control groups combined. Compared with the passive control group, the average QALY gained from the  OT intervention was 4.0 and the cost-effectiveness ratio was US$13,784/QALY. Compared with the active control group, the average QALY gained from the OT intervention was 5.2 and the cost-effectiveness ratio was US$7,820/QALY. | 91 |
| Hendriks et al. (2008)  Netherlands | To report on the economic evaluation of a multidisciplinary fall prevention program. Assess whether this program would be preferable to usual care in the Netherlands when assessed from a societal perspective in terms of costs and effects. | - older adult - frailty | - fall prevention - home-based occupational therapy | No | - randomized controlled trial - cost effectiveness analysis - cost utility analysis - Perspective: societal (service costs, family & caregiver costs home modifications& out-of-pocket costs) | The mean healthcare costs were somewhat lower (not significant) in the intervention group. The intervention group showed somewhat higher (not significant) mean patient and family costs. There was no significant difference regarding generic quality of life or fall-specific quality of life, or with regard to falls or daily functioning. There were no differences in effects or costs between the groups nor in cost-effectiveness ratios. | 92 |
| Hutchinson et al. (2020)  Australia | To investigate the social return on investment of vehicle modifications for people with disability. | - adult - disability (not specified) | - vehicle modifications - assistive technology | No | - qualitative - social return on investment analysis - Perspective: societal (service costs, service user costs) | Social return on investment ratios was positive for funder and consumer investment in all five scenarios. Social return on investment calculations based on co-investment ranged from AUD$17.32 for every $1 invested (Scenario 1) to AUD$2.78 for every $1 invested (Scenario 5). Payback periods (how long it takes for investment to be paid off in accumulated social value) for funders varied more than for consumers, as consumers’ investment was relatively consistent across scenarios. Consumers’ payback periods were between 5.4-7.1 months, and funders between 3.5 weeks and 2 years 8.4 months. | N/A |
| Hwang et al. (2018)  Australia | To investigate the cost-utility of a home-based telerehabilitation program versus a traditional centre-based rehabilitation program in patients with chronic heart failure. | - older adult - cardiovascular problems | - telehealth - rehabilitation | No | - randomized controlled trial - cost utility analysis - Perspective: service costs | Total health care costs per participant were significantly lower in the telerehabilitation group during the 6 months. No significant differences in QALY were seen between the two groups. The incremental cost-effective ratio (ICER) for estimated health care costs (including estimated program costs based on full attendances and heart failure readmission costs) was -AUD$5,408 per QALY gained. The ICER for all-cause health care costs (including actual program costs, and aggregated costs of all-cause emergency visits, hospital readmissions and day procedures) was -AUD$82,536 per QALY gained. | 100 |
| Ikiugu & Anderson (2007)  United States | To demonstrate the cost-effectiveness of using the Instrumentalism in Occupational Therapy conceptual practice model as a guide for intervention to assist teenagers with emotional and behavioral disorders transition successfully into adulthood. | - adolescent - mental health condition | mental health intervention | Yes | - pre-test/post-test - cost effectiveness analysis - Perspective: service costs | As it was not possible to convert the positive effects of intervention into monetary values, the cost-effectiveness analysis was calculated as a ratio of dollars invested in the program to the number of effects. There was at least one positive effect of intervention for every US$162.88 invested in the program. | 58 |
| Irvine et al. (2010)  United Kingdom | To conduct a cost effectiveness analysis of the provision of falls prevention to people identified by screening. | - older adult - not specified | fall prevention | No | - randomized controlled trial - cost effectiveness analysis - Perspective: service costs | The incremental falls prevented per person/year was 0.17 (from 2.24 to 2.07). The falls prevention programme was more costly than usual care, yet neither incremental costs nor differences in effectiveness were significant. The incremental cost per fall averted was £3,320. | 92 |
| Isarunuwatchai et al. (2017)  Canada | Using cost and effectiveness data from Markle-Reid et al. (2010), a secondary data analysis was conducted: 1) to determine the cost-effectiveness of a multifactorial fall prevention intervention compared to usual home care for community-dwelling older adults, and at risk of falling; and 2) to examine the influence of age on the cost-effectiveness of the intervention. | - older adult - not specified | fall prevention | No | - randomized controlled trial - cost effectiveness analysis - Perspective: societal (service costs, service user out-of-pocket expenses) | For the total sample, the fall prevention intervention was not cost-effective compared to usual care, regardless of a decision-maker’s willingness-to-pay (WTP) to prevent falls. For the 75-84 years group, the intervention was cost-effective with a WTP to prevent 1 fall of at least CDN$25,000. Conversely, for the 85+ years group, the intervention was cost-effective at WTP < CDN$5000 to prevent falls. | 95 |
| Jenkyn et al. (2012)  Canada | To examine the cost-effectiveness of a multifactorial falls prevention program and estimate the trade-off between the extra costs of such a program and the additional reduction of unintentional falls. | - older adult - not specified | fall prevention | No | - randomized controlled trial - cost effectiveness analysis - Perspective: societal (service costs, service user out-of-pocket costs & time costs) | Overall, the program was not cost-effective; however, the cost-effectiveness varied by region after correcting for demographic characteristics. Referral costs for the intervention were larger than the usual-care group. For both groups, the most heavily used services were family physician visits and emergency room visits. Apart from specialist visits where the intervention group had significantly higher mean costs, no significant differences between other mean health service utilisation costs were observed. However, hospitalisation costs for the intervention group were nearly three times (significant difference) that of the usual-care group. Participants in the intervention group incurred 2x total costs (significant difference) compared to the usual care group; this difference is due to higher hospitalization cost and higher specialist costs. The overall incremental cost-effectiveness ratio is equal to CDN$122,110/fall prevented. | 100 |
| Jutkowitz et al. (2012)  United States | To estimate the cost effectiveness of implementing Advancing Better Living for Elders from the perspective of a homecare agency. | - older adult - not specified | home-based occupational therapy | No | - randomized controlled trial - cost effectiveness analysis - Perspective: service costs | Under the assumptions of Model 1, the incremental cost-effectiveness ratio (ICER) (cost per one additional year of life) was US$13,179 and under the assumptions of Model 2, the ICER is US$14,800. Under the assumptions of Model 1, ABLE is cost effective greater than 50% of the time as long as a purchaser is willing to pay more than US$13,000 for one additional year of life. Under the assumptions of Model 2, ABLE is cost effective greater than 50% of the time as long as the purchaser is willing to pay more than US$14,800 for one additional year of life. | 93 |
| Kehusmaa et al. (2010)  Finland | To evaluate the cost-effectiveness of the Social Insurance Institution of Finland (SII) rehabilitation programme to support older persons to enable them to live independently at home for as long as possible. | - older adult - frailty | - group intervention - home assessment - occupation-focused | No | - randomized controlled trial - cost effectiveness analysis - Perspective: societal (only includes broad range of potential service costs) | The mean incremental cost of adding rehabilitation to standard care was €3111 per person. The incremental cost-effectiveness ratio for the Functional Independence Measure (FIM) did not show any clinically significant change, and the rehabilitation was more costly than standard care. A cost-effectiveness acceptability curve suggests that if decision-makers were willing to pay €4000 for a 1-point improvement in FIM, the rehabilitation would be cost-effective with 70% certainty. Certainty increases to 83% of the threshold value of willingness to pay is raised to €6000. | 79 |
| Kennedy et al. (2007)  Ireland | To compare inpatient and outpatient rehabilitation for patients with active rheumatoid arthritis from clinical and cost perspectives. | - adult/older adult - arthritis | - inpatient rehabilitation - rehabilitation | No | - randomized controlled trial - cost analysis - Perspective: societal (service costs, service user costs) | No sustained significant differences were detected between the two groups for the primary or secondary measures at the end of treatment or at follow-up. Total inpatient costs (€81,590) were more than three times higher than total outpatient costs (€25,450). The high inpatient costs were mainly attributable to salary costs (approximately 50% of total inpatient costs). | 72 |
| Khiaocharoen et al. (2012)  Thailand | To evaluate the cost-utility of rehabilitation for patients with stroke under Thai settings. | - adult/older adult - stroke | inpatient rehabilitation | No | - prospective observational cohort study - cost effectiveness analysis - Perspective: societal (service costs, service user direct and indirect costs, caregiver costs) | When only the government or hospital perspective was compared, the incremental cost per QALY gained from rehabilitation was 19,971 baht. When the societal perspective was compared, the incremental cost per QALY gained from rehabilitation was 24,571 baht. The rehabilitation group was more cost-effective in terms of QALY gained. When informal care costs or patient costs were excluded, the result was even more cost-effective. The rehabilitation costs under the government perspective at 50,000 baht per QALY gained would have an 80% probability of being cost-effective. Under the societal perspective, however, to achieve 80% likelihood of being cost-effective, the cost of rehabilitation would be higher than 70,000 baht per QALY gained. | 67 |
| Kiekens et al. (2011)  Belgium | To assess the current financial rehabilitation agreements and the organization and financing of musculoskeletal and neurological rehabilitation in Belgium and to make recommendations for improvement. | - adult/older adult - spinal cord injury | rehabilitation | No | - description - cost description - Perspective: service costs | For all reimbursement systems, the theoretical costs are significantly higher than the revenues. The hypothetical budget needed in 2007 would be approximately €5 million, which is in sharp contrast with the estimated costs borne by the centers of around €8 million. | 35 |
| Killaspy et al. (2016)  United Kingdom | To investigate, prospectively, outcomes and costs for patients of better quality inpatient mental health rehabilitation services and to identify the components of care associated with clinical outcomes. | - adult - mental health condition | mental health intervention | No | - prospective observational cohort study - cost analysis - Perspective: service costs | Service quality was not associated with patients’ social function or length of admission (median 16 months) at 12 months, but most patients were successfully discharged (56 %) or ready for discharge (14 %), with associated reductions in the costs of care. Factors associated with successful discharge were the recovery orientation of the service, and patients’ activity and  social skills at recruitment. There was a significant reduction in the service costs over the time horizon of the study, which was largely due to a reduction in nursing costs. Quality of care was not associated with costs of care when adjusted for service user age, gender, and social functioning. | 39 |
| Kim et al. (2018)  South Korea | To identify rehabilitation treatment cost according to age by using National Health Information Database (South Korea) for patients with cerebral palsy and investigate the change over time. | - children/adolescent/adult - cerebral palsy | rehabilitation | No | - retrospective observational design - cost description - Perspective: service costs | While the number of recently born children requiring rehabilitation treatment decreased, the number  of patients requiring this treatment in other age groups gradually increased. Annual physical therapy, OT, hydrotherapy, and botulinum toxin injection treatment costs/person increased. On the other hand, the number of orthopedic surgeries and selective dorsal rhizotomy performed decreased. The total cost of rehabilitation therapy (inpatient and outpatient) increased significantly for all age groups; it was significantly higher in the 0-6 years and 19 years and older age group compared to the 7-18 years group. | 59 |
| Lahtinen et al. (2017)  Finland | To report direct cost estimates, total costs of post-hip fracture treatment during one year after fracture and cost-effectiveness of different rehabilitations. | - adult/older adult - orthopedic | - activities of daily living - inpatient rehabilitation - mobility | No | - randomized controlled trial - cost description - cost effectiveness analysis - Perspective: societal (service costs, service user transport costs, caregiver help costs) | Control rehabilitation was significantly less expensive than physical and geriatric rehabilitation. Total institutional care costs (primary treatment, rehabilitation, and post-rehabilitation hospital care) were significantly lower for control than geriatric rehabilitation but did not differ between control and physical rehabilitation or between physical and geriatric rehabilitation. Costs of help from relatives (estimated as 30%, 50% and 100% of a home aid’s salary) with physical rehabilitation were lower than control but higher than geriatric rehabilitation. Total hip fracture treatment costs were significantly lower with physical than control rehabilitation at 50% and 100% of salary. | 77 |
| Lambert et al. (2010)  United Kingdom | To assess the cost effectiveness of an occupational therapy-led lifestyle approach to treating panic disorder in primary care compared with routine general practitioner’s care. | - adult - mental health condition | lifestyle intervention | Yes | - randomized contrelled trial - cost effectiveness analysis - Perspective: service costs | At 5 months the lifestyle intervention was far more costly than routine General Practitioner care, but it was also more clinically effective. However, significant between-group differences at 5 months were not sustained at 10 months. At 5 and 10 months, both groups experienced similar QALY gains; although the gains experienced in the lifestyle arm were greater, the mean differences were not significant. | 82 |
| Larsen et al. (2009)  Denmark | To compare the cost-effectiveness of an accelerated perioperative care and rehabilitation protocol with that of a more standard protocol for patients treated with total hip and knee arthroplasty. | - older adult - orthopedic | - hospital discharge - inpatient rehabilitation | No | - randomized controlled trial - cost effectiveness analysis - Perspective: societal (service costs, service user productivity losses) | The accelerated protocol was significantly less expensive than the standard protocol, with an average reduction in cost of 18,880 Danish kroner. Patients treated with the accelerated protocol following hip arthroplasty had an additional average gain of 0.08 QALY compared with the patients who received the standard protocol; this led to a 98% dominance of the accelerated protocol over the standard protocol. No significant or clinically relevant difference in the numbers of QALYs associated with the two protocols was observed for the patients treated with knee arthroplasty. | 100 |
| Lewin et al. (2014)  Australia | To compare the health and aged care service use and costs of older home-care clients over a two year period. | - older adult - not specified | home-based occupational therapy | No | - randomized controlled trial - cost analysis - Perspective: service costs | Restorative (reablement homecare services) clients used fewer homecare hours, had lower total homecare costs and were less likely to be approved for a higher level of aged care during follow-up. They were also less likely to have presented at an emergency department or have had an unplanned hospital admission. Additionally, the aggregated health and homecare costs of the restorative clients were lower by a factor of 0.83 over the 2-year follow-up. | 58 |
| Li et al. (2019)  Taiwan | To explore the relationship between outpatient rehabilitation care utilization and the corresponding medical costs of 3- to 12-year-old children with autism spectrum condition. | - children - autism spectrum disorder | rehabilitation | No | - retrospective chart review - cost description - Perspective: service costs | In all years, compared with the non-Autism Spectrum Condition (ASC) group, medical expenditure in the ASC group was almost 3 times higher (significant difference), and rehabilitation utilization was more widely distributed in the ASC group (significant). The average rehabilitation resource utilization was relatively higher among school-aged children; however, over time, the proportion of preschool-aged children increased to almost equal that of school-age children. Patients with ASC were less likely to use rehabilitation resources at medical centres. Elements outlined to be associated with expenditure related to rehabilitation (e.g., demographics as well as properties of relevant hospital, year of resource utilization) significantly influenced medical expenditure. | 74 |
| Li et al. (2006)  Canada | To estimate the incremental cost-effectiveness of services from a primary therapist compared with traditional physical therapists and/or occupational therapists for managing rheumatoid arthritis, from the societal perspective. | - adult/older adult - arthritis | - activities of daily living - assistive technology - home-based occupational therapy - orthoses | No | - randomized controlled trial - cost effectiveness analysis - Perspective: societal (service costs, service user & caregiver productivity losses) | Although the mean frequency of rehabilitation treatment in the primary therapist model (PTM) group was 3.4 visits, as compared with 5.3 visits for the traditional therapy model (TTM) group, because the unit cost for visiting a primary therapist was substantially more expensive, the mean treatment costs were significantly higher in the PTM group. The average number of rehabilitation visits was considerably lower in the PTM group because some patients in the TTM group received treatment from both a physical therapist and an OT. Drug costs accounted for more than half of the direct costs. Indirect costs (e.g., time lost from paid employment due to the treatment; time lost from doing chores due to health problems) accounted for 41% of societal costs in both groups. From a societal perspective, the PTM generated higher QALYs and resulted in a higher mean cost compared with the TTM in 6 months, although differences were not statistically significant. The estimated incremental cost-effectiveness ratio was CDN$13,700 per QALY gained. | 100 |
| Loisel et al. (2002)  Canada | To test the long-term cost-benefit and cost-effectiveness of the Sherbrooke model of management of subacute occupational back pain, combining an occupational and a clinical rehabilitation intervention. | - adult - pain | return-to-work intervention | No | - randomized controlled trial - cost benefit analysis - cost effectiveness analysis - Perspective: service costs | Over the course of the total follow up period (mean 6.4 years), all experimental interventions were cost-beneficial with savings in the Sherbrooke model arm moderately higher than those in the clinical and the occupational arms, although the differences were not significant. All experimental interventions saved days-on-full benefits when compared to the standard care arm. | 100 |
| Lord et al. (2013)  United States | To create a financial model capable of analyzing the net savings or costs associated with a U.S. hospital implementing an intensive care unit early rehabilitation program. | - children/adolescent/adult/older adult - not specified | rehabilitation | No | - decision analytic model - cost minimization analysis - Perspective: service costs | Net cost savings generated in the example scenario, with 900 annual admissions and actual length of stay reductions of 22% and 19% for the ICU and floor, respectively, were US$817,836. Sensitivity analyses, which used conservative- and best-case scenarios for length of stay reductions and varied the per-day ICU and floor costs, across ICUs with 200–2,000 annual admissions, yielded financial projections ranging from –US$87,611 (net cost) to US$3,763,149 (net savings). Of the 24 scenarios included in the sensitivity analyses, 20 (83%)  demonstrated net savings, with a relatively small net cost occurring in the remaining 4 scenarios, mostly when simultaneously combining the most conservative assumptions. | 65 |
| Louw et al. (2020)  South Africa | To explore the economic value of rehabilitation to South Africa, using a costed example of cerebrovascular accident rehabilitation. | - adult - stroke | - rehabilitation - return-to-work intervention | No | - description - cost benefit analysis - Perspective: societal (service costs, return-to work rate) | Even if “usual” stroke rehabilitation was provided, at the end of 5 years, the government would save at least R156.5 million. The costs of delivering the individualized intervention were estimated at about R5633/patient. Combining the survival rate, the return‐to‐work rate, and the cost of the programme, the work intervention programme could result in a net saving of R1486.0 million over 5 years. As well, stroke survivors who returned to work after rehabilitation may have fewer downstream health problems, meaning fewer contacts with the health care sector; the programme would thus be cost effective from the perspectives of stroke sufferers, families, employers, and government. | 52 |
| Markle-Reid et al. (2010)  Canada | To measure the effectiveness of a 6-month multifactorial, interdisciplinary team approach to fall prevention and on fall risk factors, and the associated costs, compared with usual home care services. | - older adult - not specified | fall prevention | No | - randomized controlled trial - cost description - Perspective: societal (service costs, service user out-of-pocket costs) | The mean 6-month costs of use for all types of health services decreased overall by 78.3 %. The change in total per-person direct costs of use of health services did not differ between the two groups. The interdisciplinary group had higher per-person costs of use of registered dietitians, OTs and supplies. Although these increased costs were offset by lower costs of use of dentists, and lower costs of use of surgeons, chiropractors, psychologists, optometrists, podiatrists, and prescription medications compared with the usual-home-care group, these changes were not significant. There was no difference between groups in use of any other type of health service, including acute hospitalisation for a fall. The total per-person direct costs of use of health services did not differ between the participants in the sub-groups compared to similar participants in the control group. | 81 |
| Merkesdal & Mau (2005)  Germany | To assess societal costs-of-illness and their changes in the year prior to and after outpatient rehabilitation in initially gainfully employed persons with low back pain and to identify predictors (cost drivers) for high overall costs in the year after the intervention. | - adult - pain - orthopedic | rehabilitation | No | - prospective before-and-after - cost analysis - Perspective: societal (service costs, service user productivity losses) | Costs due to sick leave periods represent the major component (83%) of overall costs prior to, and 12 months following, outpatient rehabilitation (OPR). The comparison of costs 12 months before and after OPR revealed a significant reduction from h8050 to h3200 per person, primarily due to decreasing sick leave costs and the reduction of costs related to inpatient treatment. The prediction analysis revealed that patients with limited functional abilities, with problems due to strenuous labour,  with low expectations in terms of possible improvement after OPR, with a high pain score or with limited satisfaction with working colleagues have a significantly higher risk for  costs exceeding h2200 after OPR. | 77 |
| Miller et al. (2005)  United Kingdom | To measure the cost-effectiveness of an early discharge and rehabilitation service (EDRS) in Nottingham (United Kingdom). | - older adult - orthopedic - stroke - cardiovascular problems - mixed neurological conditions - pulmonary disease | - activities of daily living - home-based occupational therapy | No | - randomized controlled trial - cost analysis - cost effectiveness analysis - Perspective: service costs | The EDRS reduced the mean length of hospital stay by 9 days, using an average of 22 visits. The total cost per case was £1,727 lower in the early discharge and rehabilitation service (EDRS) group, although cost data were positively skewed. The cost savings were largely due to reduced length of hospital stay and reduced use of day hospitals. At 12 months, the mean untransformed total cost for the EDRS was £8,361 compared to £10,088 for usual care, a significant saving of £1,727. Cost-effectiveness acceptability curves showed a high probability that the EDRS was cost effective across a range of monetary values for a QALY. | 87 |
| Mortimer et al. (2019)  Australia | To evaluate the cost-effectiveness of structured activities of daily living retraining during posttraumatic amnesia plus treatment as usual (TAU) vs TAU alone for inpatient rehabilitation following severe traumatic brain injury. | - adult/older adult - brain injury | - activities of daily living - occupation-focused | No | - randomized controlled trial - cost effectiveness analysis - Perspective: service costs | Structured ADL retraining during posttraumatic amnesia (PTA) significantly increased functional independence at PTA emergence and hospital discharge. Even in the most pessimistic scenario, structured ADL retraining was cost-saving as compared to treatment as usual (TAU). Together, these results suggest that structured ADL retraining dominates (less costly but as effective) TAU when effectiveness is evaluated at PTA emergence and hospital discharge. | 97 |
| Nagayama et al. (2021)  Japan | To examine the association between intensive rehabilitation for patients with subacute stroke and medical costs and the readmission ratio during the 1 year after discharge. | - adult - stroke | - inpatient rehabilitation - rehabilitation | No | - retrospective chart review - cost description - Perspective: service costs | In the acute phase, there were no significant differences between groups in daily rehabilitation time, total rehabilitation time, length of hospital stay, and total hospital cost. In the convalescent rehabilitation unit, the high-intensity rehabilitation group exhibited significantly higher values than did the low-intensity rehabilitation group for daily rehabilitation time, total rehabilitation time, and total medical costs. There was no significant difference in medical costs during the year after discharge nor in the readmission ratio. | 88 |
| Nagayama et al. (2017)  Japan | To determine the cost effectiveness of the occupation-based approach using the Aid for Decision-Making in Occupation Choice (ADOC) for subacute stroke patients compared with the impairment-based approach. | - adult/older adult - stroke | - inpatient rehabilitation - rehabilitation - occupation-focused | Yes | - randomized controlled trial - cost effectiveness analysis - Perspective: service costs | For adjusted QALYs, the intervention group was significantly higher than the control group. The cost of one QALY gain was JPY 12.7 million for the intervention group versus JPY 13.8 million for the control group. The total costs did not significantly differ. The Aid for Decision-Making in Occupation Choice (ADOC) was found to be more effective and less costly. The probability of the occupation-based approach being cost effective compared to the impairment-based approach was estimated to be 65.3%, with a willingness-to-pay of JPY 5 million/QALY. | 100 |
| Nagayama et al. (2016)  Japan | To compare the effectiveness, including cost-effectiveness, of the occupation-based approach versus the impairment-based approach for older residents and to determine the feasibility of conducting a large cluster randomized controlled trial in this setting. | - older adult - stroke - orthopedic - cognitive decline - frailty | - activities of daily living - occupation-focused | Yes | - randomized controlled trial - cost effectiveness analysis - Perspective: service costs | There were no significant differences between the groups for the total costs. The cost-effectiveness ratios were US$61,743.20 /QALY and US$3,347.20/Barthel Index (BI) change in the Aid for Decision-making in Occupation Choice (ADOC) group and US$52,902.60/QALY and US$23,925.30/BI change in the control group. The change in BI score was the only variable with a significant difference between the groups; the incremental cost-effectiveness ratio, which was calculated using the change in BI score, was US$63.10. | 66 |
| Norrefalk et al. (2008)  Sweden | To evaluate the economic consequences of an 8-week, work-related, multiprofessional medical rehabilitation programme for patients on long-term sick-leave with persistent musculoskeletal-related pain. | - adult - pain | return-to-work intervention | No | - matched-pairs design - cost description - Perspective: service costs | The benefit of the programme was estimated to be €3,799–7,515 per treated patient and year. The total cost of the programme was estimated to be €5,406 per patient. Based on these figures the total cost of the programme, including  costs for patients remaining on sick leave, had been recovered when the successfully rehabilitated patients had worked for 9–17 months. Any additional work after that yielded net economic benefits. | 45 |
| O’Connor et al. (2011)  United Kingdom | To examine the reduction in care costs that can be achieved by a goal-orientated multidisciplinary inpatient rehabilitation programme following acute stroke management. | - adult - stroke | inpatient rehabilitation | No | - prospective before-and-after - cost analysis - Perspective: service costs | The median calculated weekly cost of care for these patients reduced significantly from £1,900 to £1,100. The total annualised care costs were reduced from £3,358,056 to £1,807,208, which represents a potential saving of £1,550,848 over one year. The median time to repay (theoretical payback time for the cost of participation in the rehabilitation programme) rehabilitation costs was 21 weeks. Savings occurred in those with moderate and severe disability, and they have the potential to continue to accrue for over 12 years. | 38 |
| Padwal et al. (2012)  Canada | To examine the impact of severe obesity on length of stay, functional status change, and inpatient costs in a population-based, publicly funded, tertiary care regional Canadian rehabilitation center. | - adult/older adult - obesity | hospital discharge | No | - retrospective observational design - cost description - Perspective: service costs | Severely obese subjects had a significantly greater mean overall length of stay (LOS), mean rehabilitation LOS, and waiting-transfer-of-service LOS compared to controls. Although LOS outcomes were consistently higher in the severely obese, the results were statistically significant only in the orthopedic group. Severe obesity was a significant independent predictor of greater overall LOS and rehabilitation LOS. Total daily hospital costs were CDN$115,822 in the severely obese compared to CDN$43,969 in controls. | 42 |
| Peeters et al. (2011)  Netherlands | To evaluate the cost-effectiveness of multifactorial evaluation and treatment of fall risk factors compared to usual care in community-dwelling older persons at high risk of recurrent falling. | - older adult - not specified | fall prevention | No | - randomized controlled trial - cost effectiveness analysis - Perspective: societal (service costs, service user and caregiver costs) | The intervention did not reduce the fall risk as compared with usual care during 1 year of follow-up. The average costs made from a societal perspective in persons with a high risk of recurrent falling who received the multifactorial intervention was €7,740 in 1 year, which was €902 higher than in the control group that received usual care. Cost-effectiveness planes and acceptability curves indicated that multifactorial evaluation and treatment of fall risk factors was not cost-effective compared with usual care. | 97 |
| Pergolotti et al. (2018)  United States | To describe the cost of occupational therapy by provider, insurance status, and geographic region and the number of visits allowed and out-of-pocket costs under proposed therapy caps. | - adult/older adult - not specified | - activities of daily living - occupation-focused - mobility - rehabilitation - therapeutic exercise | Yes | - retrospective chart review - cost description - Perspective: societal (service costs, service user out-of-pocket costs) | Wide variation exists in potential patient out-of-pocket costs for OT services based on insurance coverage and state. Patients without insurance pay a premium. | 70 |
| Pizzi et al.  (2022)  United States | The main goal of this cost benefit analysis was to determine if the Care of Persons with Dementia in their Environments (COPE) program delivered with home- and community-based service (HCBS) compared to HCBS alone (usual care). Secondarily, the study sought to examine a potential payment model whereby COPE is a covered service under Connecticut HCBS, based on its fit within monthly spending limits. Finally, the study examined whether caregivers’ willingness to pay (WTP) for COPE falls within Connecticut Home Care Program for Elders (CHCPE) cost-sharing levels. | - older adult - cognitive decline | - activity-based - fall prevention - home adaptation - home assessment - home-based occupational therapy - occupation-focused | Yes | - randomized controlled trial - cost benefit analysis - Perspective: societal (service costs, caregiver time) | Per-dyad mean cost savings at 12 months were US$2354 for those who received COPE with a mean (non-significant) difference of −US$6 667 versus HCBS alone, representing a cost saving.  OT/APN time was the costliest component, followed by travel time, laboratory testing, and formal staff training.  The COPE group had a mean per-dyad (non-significant) difference of −US$8 867, representing a health care services cost saving for COPE. | 100 |
| Pizzi et al.  (2023) | To investigate the costs of delivering the Tailored Activity Program (TAP) and cost savings from two perspectives (health sector  and societal) for people living with dementia (PLWD) and their caregivers (dyads) compared to attention control (AC) using data from a randomized controlled trial. | - adult/older adult - cognitive decline | - activities of daily living - activity-based - home-based occupational therapy - occupation-focused | Yes | - randomized controlled trial - cost analysis - Perspective : societal (service costs, caregiver time) | The total intervention cost of TAP was US$1707/dyad versus US$864/dyad for AC.  Total costs over 6 months for TAP dyads as compared to AC were US$1299 less from the healthcare perspective, and US$761less from the societal perspective. | 86.9 |
| Pizzo et al.  (2022) | The main aim of this study was to assess the costs, outcomes and the cost-utility of the COTiD-UK intervention compared to Treatment as usual (TAU), using data from the VALID RCT. | - adult/older adult - cognitive decline | - home assessment - home-based occupational therapy - occupation-focused | Yes | - randomized controlled trial - cost utility analysis - Perspective : societal (service costs, service user productivity losses & out-of-pocket costs, caregiver productivity, out-of-pocket, & transport costs) | Total Bristol Activities of Daily Living Scale (BADLS) score at 26 weeks did not differ significantly between groups. Secondary outcomes (cognition, quality of life, assistance needed with ADL, mood, carer sense of competence and mood) did not differ between the groups. In total, 91% of the activity-based goals set by the pairs taking part in the COTiD-UK intervention were fully or partially achieved by the final session. There was a significant difference in costs between COTiD-UK and TAU, but no significant difference in outcomes (mean QALYs gained).  The Incremental Net Monetary Benefit (INMB) for COTiD-UK versus TAU was negative at a maximum willingness to pay for a QALY of £20000. | 100 |
| Puolakka et al. (2007)  Finland | To explore the cost of the statutory inpatient rehabilitation system in Finland and its impact on the functional and work capacity of patients with early rheumatoid arthritis. | - adult/older adult - arthritis | - group intervention - inpatient rehabilitation - return-to-work intervention | No | - randomized controlled trial - cost analysis - Perspective:service costs | Of the patients in the REHAB group, 35% ended with a rheumatoid arthritis (RA)-related disability pension compared to 20% of the no-REHAB group. Being in the REHAB group was associated with increased RA-related retirement; the sex- and age-adjusted hazard ratio was 2.15. Inpatient rehabilitation had no impact on working ability in terms of lost productivity nor any influence on patients’ functional capacity. | 37 |
| Radford et al. (2013)  United Kingdom | To determine whether a traumatic brain injury (TBI) specialist vocational rehabilitation (VR) intervention (TBI VR) delivered by an occupational therapist as part of a specialist TBI team approach to care was more effective at supporting work return and retention 12 months after injury in people with TBI than usual care (UC) and to explore the feasibility of economic data collection and evaluation for a definitive trial. | - Adolescent/adult/older adult - Brain injury | - return-to-work intervention | Yes | - prospective observational cohort study - cost effectiveness analysis/cost utility analysis - Perspective: societal (service costs, service user and caregiver lost wages & out-of-pocket costs) | When the broader perspective for cost was used, it cost substantially less to return a person to work with TBI-VR than UC. From a societal perspective, TBI-VR was always cheaper and more effective. | 100 |
| Radford et al. (2018)  United Kingdom | To determine the feasibility of conducting a multicentre randomised controlled trial comparing the clinical effectiveness and cost-effectiveness of Early Specialist Traumatic brain injury Vocational Rehabilitation  (ESTVR), delivered by National Health Service (NHS) occupational therapists (OTs) in addition to usual NHS rehabilitation, with usual  NHS rehabilitation alone [usual care (UC)] on work (work return and job retention) and health outcomes at 12 months post injury. | - Brain injury | - return-to-work intervention | Yes | - randomized controlled trial - Cost effectiveness analysis - Perspective : service costs | The vocational rehabilitation (VR) intervention group was, on average, cheaper than the usual care (UC) group; however, in the VR group, fewer people returned to, or retained, work. The net monetary benefit (NMB) for the VR intervention group, when considering the intervention costs in the analysis, was £18.64 when using a £20,000 willingness-to pay (WTP) per QALY threshold and –£487.36 at the WTP threshold of £30,000 per QALY. At a WTP threshold of £20,000, the NMB is above zero, which indicates that TBI may be cost-effective at currently accepted thresholds. The probability of VR being cost-effective at a WTP threshold of £20,000 (£30,000) per QALY was 47.00%. The expected value of perfect information (EVPI) was £3077.17 (£3741.49) per participant at a threshold ICER of £20,000 (£30,000). It is feasible to assess the cost-effectiveness of vocational rehabilitation (VR). | 100 |
| Rahja et al. (2020)  Australia | To identify the costs and benefits of implementing the Care of People with dementia in their environments (COPE) program in the existing Australian health context from different perspectives. The secondary aim was to assist policy makers in appraising the program potential for more widespread adoption. | - older adult - cognitive decline | - activities of daily living - home-based occupational therapy - occupation-focused | Yes | - pre-test/post-test - cost benefit analysis - Perspective : societal (service costs, service user and caregiver costs) | A reduction in longer term and hospital-based healthcare use, as well as healthcare services used in the community. An increase in services accessed at home (e.g., personal care). While the amount of time caregivers spent supervising or assisting the person with dementia in ADL (e.g., showering, dressing, eating) was reduced, the caregivers reported spending more time assisting the person with dementia in independent ADL (e.g., shopping, cleaning, community participation). A reduction in time spent away from paid employment. The main contributors to the program cost were those related to the cost of therapists delivering and participants paying for the intervention. People with dementia and their caregivers endured most of the ongoing costs. Societal gain: most prominent from reduced use of hospital and long-term care services, and reduced healthcare service use in the community. Other noticeable gains included reduction in time spent caregiving and away from paid employment, as well as improvement in the assumed quality of life for the participant dyads. The Australian health and social care system benefits the most out of the program implementation and adoption. | 70.1 |
| Rasmussen et al. (2016)  Denmark | To evaluate if home-based rehabilitation of inpatients with Stroke improved outcome compared to standard care. | - older adult | - activities of daily living - community-based - home-based occupational therapy - occupation-focused - pre-discharge home visits - rehabilitation - transfers | No | - randomized controlled trial - cost analysis - Perspective : service costs | The average total costs of an intervention patient corresponded to US$ 54,118.00 compared to US$ 54,242.00 for each control patient, corresponding to savings of US$ 124 or 0.2 % in favour of home-based rehabilitation. | 43.4 |
| Roderick et al. (2001)  United Kingdom | To present a comparison of the cost-effectiveness of a new domiciliary rehabilitation service for stroke patients with rehabilitation in geriatric day hospitals. | - adult/older adult - stroke | - home-based occupational therapy - rehabilitation | No | - randomized controlled trial - cost effectiveness analysis - Perspective : service costs | Total cost per patient did not differ significantly between the two groups, with reduced health service costs in the domiciliary group offset by higher social service costs. | 63.6 |
| Rodgers et al. (2019)  United Kingdom | To determine the clinical and cost effectiveness of an extended stroke rehabilitation service (EXTRAS). | - adult/older adult - stroke | - activities of daily living - cognitive - mental health intervention - mobility | No | - randomized controlled trial - cost effectiveness analysis - Perspective: service costs | Over 24 months, the mean cost of resource utilization was lower in the intervention group. The cost savings were predominantly in social care rather than health care. Patients in the intervention group experienced 0.07 additional QALYs. There is a 68% chance that the extended stroke rehabilitation service (EXTRAS) is cost saving. At the current National Health Service standard of willingness to pay £20 000 per QALY, there was a 90% probability that the EXTRAS intervention could be considered cost-effective. | 52 |
| Rodgers et al. (2003)  United Kingdom | To determine whether an early increased-intensity upper limb therapy programme, provided jointly by an occupational therapist and a physiotherapist, following acute stroke improves outcome. | - older adult - cerebrovascular accident | - inpatient rehabilitation - rehabilitation, - therapeutic exercise | No | - randomized controlled trial - cost analysis - Perspective: service costs | There were no significant differences between the groups in total health and social care costs within the 6 months after stroke. | 50.5 |
| Rogers et al. (2016)  United States | To provide information that hospital executives can use to make efficient resource allocation decisions. | - older adult - cardiovascular problems | hospital discharge | Yes | - retrospective chart review - cost analysis - Perspective: service costs | OT was the only category where additional spending had a significant association with lower readmission rates for all 3 medical conditions (heart failure, pneumonia, acute myocardial infarction). Spending on emergency room and cardiology services had the second most consistent significant association with lower readmissions, but these results were not significant for all three outcome measures. | 82 |
| Ruchlin et al. (2001)  United States | To assess the cost savings associated with a patient education and high-intensity strength intervention to improve rehabilitation after hip fracture. | - older adult - orthopedic | rehabilitation | No | - randomized controlled trial - cost analysis - cost benefit analysis - Perspective: societal (service costs, caregiver non-paid assistance) | The intervention group used more physician care, diagnostic tests, emergency department visits, nursing home and rehabilitation facility admissions than the control group. The control group used more physical and OT sessions and hours of both formal and informal help than the intervention group members. With the exception of rehabilitation and nursing home care, the cost patterns mirror these utilization patterns. The intervention group displayed a higher cost for prescription drugs than the control group members, but lower equipment costs. The average cost of both formal and informal care was higher in the control group than in the intervention group; members of the control group also incurred higher transportation costs. None of the actual utilization and cost category differences were significant. However, while it cannot be concluded that intervention patients used fewer resources than control patients, they did not use more care. All the benefit/cost ratios exceeded 4.5, indicating that benefits exceed costs. | 86 |
| Sackley et al. (2016)  United Kingdom | To conduct a Phase III RCT to evaluate the effects of a targeted 3-month course of occupational therapy (with provision of adaptive equipment, minor environmental adaptations and staff education) for people with stroke sequelae living in care homes. | - older adult - stroke | - occupation-focused - assistive technology - mobility - transfers | Yes | - randomized controlled trial - cost utility analysis - Perspective: service costs | The mean incremental QALY gain was 0.009, with wide confidence intervals. Neither the mean incremental cost nor the mean incremental QALYs reached significance. The incremental cost effectiveness ratio was estimated to be £49,825/QALY, suggesting less favourable cost effectiveness. The outcomes were virtually equivalent in both arms. Costs were higher in the intervention arm. The intervention did not lead to a reduction in health resource use from other sources. | 94 |
| Sahota et al. (2017)  United Kingdom | To examine the clinical effectiveness and cost-effectiveness of community/intermediate care services such as the Community In-Reach and Care Transition service, compared to standard current UK practice, the traditional hospital-based rehabilitation service. | - older adult - frailty | - hospital discharge - home assessment - home-based occupational therapy | No | - randomized controlled trial - cost effectiveness analysis - Perspective: service costs | There was no significant difference in length of stay between the Community In-reach Rehabilitation and Care Transition (CIRACT) and traditional hospital-based rehabilitation (THB-Rehab) service. There were no significant differences in any of the secondary outcomes between the two arms. The mean incremental cost-effectiveness ratio for CIRACT versus THB-rehab service was £2,022 per QALY. The net monetary benefit per patient per year (willingness to pay threshold at £30,000 per QALY) was £1,932, and the probability that the intervention is cost-effective was 0.91. | 56 |
| Salkeld et al. (2000)  Australia | To estimate the cost- effectiveness of one component of a multifactorial approach to falls prevention (home hazard reduction program). To estimate the size and direction of change in resource use within and between the hospital, home and community sectors. | - older adult - frailty | fall prevention | Yes | - randomized controlled trial - cost effectiveness analysis - Perspective: societal (service costs, caregiver informal care time) | The difference in total median cost between the 2 groups was not significant. However, differences between the groups are accentuated when the data are described by falls history; older persons who had fallen in the last year consumed more home and community care services and experienced higher levels of informal care. The incremental cost per fall prevented for all subjects was AUD$4,986. When median costs were used, the intervention achieved an  incremental ratio of AUD$2,853 per fall prevented for all subjects and only AUD$119 per fall prevented for subjects with a history of falls. | 83 |
| Sampson et al. (2014)  United Kingdom | To estimate the cost-effectiveness of occupational therapy home visits after stroke, as part of a feasibility study, and to demonstrate the value and methods of economic evaluation. | - adult/older adult - stroke | home assessment  hospital discharge | Yes | - randomized controlled trial - cost utility analysis - Perspective: service costs | The incremental cost-effectiveness ratio, or cost-per-QALY of home visits, was £21,987. Based on this analysis of a small sample, it is estimated that there is a 47% chance that home visits are cost-effective at a willingness-to-pay of just over £20,000 per QALY. | 93 |
| Samuelsson & Wressle (2014)  Sweden | To evaluate the effect of electric powered wheelchairs and scooters on occupational performance, social participation, and life satisfaction. To estimate the cost-benefit of the assistive device and to describe users’ experiences with the delivery process. | - adult/older adult - mobility challenges | mobility assistive device | No | - prospective before-and-after - cost benefit analysis - Perspective : societal (service costs, caregiver support time) | The estimated direct mean cost for one device [electric powered wheelchairs/ scooters (PWC/S)] during the first year was estimated to be €1188 (mean rental cost) + €281 (mean providing process cost) = €1469 per user. The societal savings based on the total cost for the assistive device (rent and provision) minus a decrease of costs for personal assistance was €6227/year for each user. Even if the decrease of assistance would be just 1 hour/week, the use of PWC/S would still be profitable for society. | 69 |
| Schene et al. (2007)  Netherlands | To determine the cost-effectiveness of the addition of occupational therapy treatment to treatment as usual for individuals with major depression. | - adult - mental health condition | - group intervention - mental health intervention - return-to-work intervention | Yes | - randomized controlled trial - cost effectiveness analysis - Perspective: societal (service costs & employer work time costs) | The addition of OT to treatment as usual (TAU): (i) did not improve depression outcome, (ii) resulted in a reduction in work-loss days during the first 18 months (albeit not for months 19-42), (iii) did not increase work stress, and  (iv) had a 75.5% probability of being more cost-effective than TAU alone. | 88 |
| Schneider et al. (2007)  United Kingdom | To report the cost of providing a full-time occupational therapist to care homes and explore the effect of such an intervention on the use of other services and associated costs. To measure the amount of occupational therapy services used by the individuals in the study, with a view to its replication, if it proved effective in alleviating depression. | - older adult - cognitive decline | group intervention  occupation-focused | Yes | - randomized controlled trial - cost minimization analysis - Perspective: service costs | The intervention group significantly increased their use of social care (social worker, day care outside the home, home care) over the research period compared to the control group, which might be explained by previously unrecognised needs being revealed by the OTs. The net cost of the intervention was, on average, £15-16 per week. For this, a resident in the intervention group received over 30 minutes of individual OT input and over an hour of group input each week. | 87 |
| Shah et al. (2020)  United States | To evaluate baseline use, the change in use, variation in prescribing patterns by region, and costs for physiotherapy and occupational therapy after common hand procedures. | - adult - hand problem | - orthoses - hand therapy - rehabilitation - therapeutic exercise | No | - retrospective chart review - cost description - Perspective: societal (service costs, service user out-of-pocket costs) | The incidence of 90-day utilization of PT and OT after hand procedures was 14.0% and increased for all procedures during the observation period with an average compound annual growth rate (CAGR) of 8.3%. Cost/therapy visit was relatively stable when adjusted for inflation, with an average CAGR of 0.63%. Patients in the northeast had a significantly higher incidence of PT/OT use than those in the south and west for all procedures except carpometacarpal arthritis. | 80 |
| Sheffield et al. (2013)  United States | To examine the economic impact of the intervention relative to traditional home care interventions provided by the agencies. | - older adult - not specified | - activities of daily living - fall prevention - home-based occupational therapy | Yes | - randomized controlled trial - cost analysis - cost description - Perspective: service costs | After the OT intervention, the OTs assessed each participant’s need for continued assistance. They recommended a reduction of 2.36 hours/week per client, or a 39% reduction relative to the existing care plan created by the social worker/case manager. If these recommendations were to be implemented, there would be significant savings potential relative to the status quo. | 27 |
| Shimada et al. (2020)  Japan | To evaluate the cost-effectiveness of adding individualized occupational therapy to group occupational therapy (GOT) as standard care versus GOT alone for prevention of rehospitalization for patients with schizophrenia. | - adult - mental health condition | mental health intervention | Yes | - randomized controlled trial - cost effectiveness analysis - Perspective: service costs | The number of patients who avoided rehospitalization was significantly higher in the group OT (GOT) + individualized OT (IOT) condition compared with the GOT alone condition. Adding IOT to GOT was associated with a 56.76% probability of being more effective at  reducing the rehospitalization rate and a 26.93% probability of being less costly than GOT alone. GOT + IOT had high  outpatient costs, but lower inpatient costs due to the lower rehospitalization rate; as a result, total medical costs were  lower than for GOT alone. | 93 |
| Sigurdsson et al. (2008)  Iceland | In this study, a comparison of total costs associated with total hip replacement was conducted between an established clinical routine and a new concept based on preoperative education and postoperative home intervention after a shortened hospitalization. | - adult/older adult - arthroplasty | - activities of daily living - early hospital discharge - home assessment - inpatient rehabilitation - mobility assistive device - therapeutic exercise | Yes | - randomized controlled trial - cost effectiveness analysis - Perspective: societal (service costs & service user contribution to costs) | There was a significant (28%) cost reduction for the study group participants, reflecting a cost-effectiveness gain of 40%. This difference is predominantly due to a shorter hospital stay and to home intervention instead of a stay at a convalescent home. The difference in inpatient hospital cost between the groups makes for more than half the total. For outpatient costs, the greatest difference between the groups is due to home intervention instead of convalescent home. | 97 |
| Sletten et al. (2015)  United States | To analyse the financial costs and benefits of treating patients with chronic pain in a comprehensive outpatient pain rehabilitation program. | - adult/older adult - pain | activities of daily living  occupation-focused | No | - retrospective chart review - cost minimization analysis - Perspective: societal (service costs & service user contribution to costs) | Medical costs decreased by 86, 68, 64, and 90% in the 3-, 6-, 12-, and 18-month post-treatment periods, respectively, when compared with the  same pretreatment periods. | 70 |
| Smeets et al. (2009)  Netherlands | To examine whether a combination of a physical training and operant-behavioral graded activity with problem solving training is cost-effective compared to either alone 1-year post-treatment, a full economic analysis alongside a randomized controlled trial was conducted. | - adult - pain | occupation-focused  rehabilitation | No | - randomized controlled trial - cost effectiveness analysis - cost utility analysis - Perspective: societal (service costs, service user direct non-healthcare costs and productivity losses) | APT (Active physical treatment-no OT involvement), followed by CT (Combination Treatment), showed higher, although not significant, total costs than GAP (Graded activity with problem solving training). Reduction of disability and gain in QALY did not differ significantly between CT and the single treatment modalities. Based on the incremental cost effectiveness  ratios and cost-effectiveness acceptability curves CT is not cost-effective. However, GAP is cost-effective regarding the reduction of disability and gain in QALY, and to a lesser degree APT is more cost-effective than CT in reducing disability. | 82 |
| Spoelstra et al. (2019)  United States | To describe the impact of a geriatric model of care on health outcomes and emergency department visits and hospitalizations in the Michigan Medicaid Home and Community Based Waiver Program (“waiver”). | - adult/older adult - not specified | - activities of daily living - fall prevention - home assessment - home-based occupational therapy | No | - matched-pairs design - cost analysis - Perspective: service costs (emergency department & hospitalisation) | No changes in the number of emergency department visits over the past 90 days were found pre- to post-CAPABLE (Community Aging in Place, Advancing Better Living for Elders), and the means were not different from the usual care comparison group. The intervention group had a reduction in hospitalizations over the past 90 days from pre- to post-CAPABLE. The post-CAPABLE mean number of hospitalizations was significantly lower than in the usual care comparison group. Among those 65 years and older, females had greater reduction in the number of hospitalizations than males, while no sex difference existed among those younger than 65 years. | 58 |
| Sritipsukho et al.  (2010)  Thailand | To compare costs and effects of a home rehabilitation program versus conventional hospital care for ischemic stroke patients in a Thai healthcare setting. | - adult/older adult - stroke | home-based occupational therapy | No | - randomized controlled trial - cost effectiveness analysis - Perspective: service costs | The cost-effectiveness ratios for the study group were lower than those of the control group. The lowest incremental cost-effectiveness ratio was for the Modified Rankin Scale (MRS) measurement, followed by those of the BI 1 (Barthel Index 1: achieving mild or no disability) and B1 2 (Barthel Index 2: achieving no disability). Sensitivity analyses regarding variations in number of patients and cost of home visits  demonstrated more cost-effectiveness than the base case. | 70 |
| Sturkenboom et al. (2015)  Netherlands | To evaluate the cost-effectiveness of the home-based Occupational Therapy in Parkinson’s Disease (OTiP) trial. | - older adult - Parkinson’s disease | home-based occupational therapy | Yes | - Randomized controlled trial - cost effectiveness analysis - Perspective: societal (service costs, service user productivity loss, caregiver time) | OT did not significantly impact total costs (patients, caregivers, patient-caregiver) compared with usual care. At a value of €40,000 per QALY gained, only the net monetary benefit of the intervention per caregiver was significant. When society is willing to pay €20.000 per QALY gained, the net benefit of the intervention for the caregiver is positive, with a probability of 95%. | 79 |
| Szanton et al. (2018)  United States | To determine whether the Community Aging in Place, Advancing Better Living for Elders (CAPABLE) program saves Medicaid more money than it costs to provide. | - older adult - chronic illness | home-based occupational therapy | No | - quasi-experimental - cost analysis - Perspective: service costs | The treatment group had: (a) an 11% lower probability of having an expenditure for any service type, (b) a lower probability of using every service type (except for home health) than the comparison group among people who incurred expenditures; (c) a significantly lower probability of using inpatient, outpatient, and specialist services and a higher probability of using home health services. The average Medicaid spending per CAPABLE participant was US$867 less/month than that of their matched comparison counterparts.  The largest differential reduction in expenditures were for inpatient care and long-term services and supports. | 62 |
| Tam et al. (2019)  Canada | To compare fast-track (FT) rehabilitation within 1 week of discharge with no FT in a single healthcare payer system. | - adult/older adult - stroke | - hospital discharge - inpatient rehabilitation - rehabilitation | No | - retrospective comparative study - cost effectiveness analysis - Perspective: service costs | The incremental cost effectiveness ratio (ICER) estimate for patients entering FT (outpatient high-intensity fast-track stroke rehabilitation program) from inpatient rehabilitation was CDN$404 ($270–620) per inpatient day saved. The ICER estimate for direct from acute care admissions was CDN$37 per day saved. At willingness-to-pay of CDN$698 (cost of one alternate level of care day in acute care awaiting rehabilitation), the probability of FT being cost-effective was 99.2 and 100% for patients from inpatients rehabilitation and acute stroke care, respectively. | 97 |
| Teng et al. (2003)  Canada | To estimate the costs associated with the early supported discharge (ESD) program compared with those of usual care. A secondary objective is to estimate the impact of the ESD program on caregiver burden. | - adult/older adult - stroke | - activities of daily living - home-based occupational therapy - rehabilitation | No | - randomized controlled trial - cost effectiveness analysis - Perspective : service costs | The total cost generated by persons assigned to the home group was lower (significant). A large proportion of this cost differential arose from readmissions, for which the usual care group generated costs more than 4x those of the home intervention group. Providing care at home was no more or less expensive for those patients with greater functional limitations than for those with less. Because the home intervention proved to more effective than usual care, reduced caregiver burden, and had lower costs, it is more cost-effective than usual care. | 78.8 |
| To et al. (2022)  United States | To quantify health care resource utilization (HCRU) and costs for people with Huntington disease (HD) in the United States, overall and by stage, and compare these with matched non-HD controls. | - adult/older adult - Huntington disease | - activities of daily living difficulties - functional difficulties - quality of life | No | - retrospective comparative study - cost analysis - Perspective : service costs | Health care resource utilization (HCRU) in 6 months post-index was significantly greater in people with Huntington disease (HD) compared with non-HD controls for all health care service categories. The mean number of HCRU per-patient-per-month for all measured healthcare services was significantly higher in people with HD compared with non-HD controls. Mean total costs for the HD cohort were twice the total costs in the non-HD cohort and were highest across all disease stages. | 55.6 |
| Toida & Takemura (2002)  Japan | To conduct a cost-benefit analysis of a community-based rehabilitation program provided by municipalities. | - adult/older adult - stroke | rehabilitation | No | - survey - cost benefit analysis - Perspective: service costs | The mean and median willingness to pay (WTP) were 441 yen and 300 yen respectively. The WTP was negatively related to the duration of functional disability, and positively related to income. The costs per capita without the cost of volunteers among 18 wards ranged from 2079-6732 yen, and those with the cost of volunteers ranged from 3289-8366 yen. Compared with the range of the costs and benefits reported, the WTP was found to be much lower, resulting in a net cost for the programme. The sensitivity analyses revealed that this was true for the range of costs tested. | Insufficient infor-mation to assess with QHES |
| Tousignant et al. (2005)  Canada | To test a cost estimation methodology in the context of rehabilitation services delivered at home for lower limb orthopedic surgery patients and to provide preliminary data on the costs. | - older adult - orthopedic | home-based occupational therapy | No | - description - cost description - Perspective: service costs | For each hour of direct time to deliver the program, nearly 2 hours were required: 0.9 hours of direct time, 0.9 hours to organize the service, do the administrative work, and 0.3 hours to travel to the patient’s home. At least CDN$2095 must be invested in home rehabilitation services to see a clinical improvement, that is, CDN$ 419 per unit of change of functional autonomy. | 55 |
| Tung et al. (2021)  Taiwan | To compare the current inpatient post-acute care (PAC) model with a novel home-based PAC model in cost-effectiveness and functional recovery for stroke patients in Taiwan. | - adult/older adult - stroke | - inpatient rehabilitation - home-based occupational therapy | No | - retrospective comparative study - cost effectiveness analysis - Perspective: service costs | The total rehabilitative cost was significantly cheaper in the home-based post-acute care (PAC) group. The cost-effectiveness is US$152.474±164.661 in the inpatient group, and US$48.184 ±35.018 in the home group (p < 0.001). Fewer rehabilitative hours per 1-point increase of Barthel Index score was noted in the home-PAC group with similar improvements in daily activities, life quality and nutrition in both groups. | 59 |
| Turner-Stokes et al. (2012)  United Kingdom | A “real-life” application is presented of the UK Rehabilitation Outcomes Collaborative dataset from two tertiary neurological rehabilitation services carrying a highly complex caseload to demonstrate how the dataset may be used to compare the cost-efficiency of different service models, and to make the case for resources to maximise efficiency and to improve patient care. | - adult/older adult - mixed neurological conditions | rehabilitation | No | - retrospective chart review - cost description - Perspective: service costs | The mean length of stay for Unit A was 1.5 times longer than Unit B, which had 85% higher levels of therapy staffing in relation to occupied bed days. Therefore, despite higher bed-day costs, Unit B was 20% more cost-efficient overall, for similar gain. | 51 |
| Van den Hout et al. (2007)  Netherlands | To estimate, from a societal perspective, the cost-utility of the program for patients with chronic rheumatic diseases at risk of job loss. | - adult - arthritis | vocational rehabilitation | No | - randomized controlled trial - cost utility analysis - Perspective: societal (service costs, service user contribution to costs, productivity losses & unpaid labour) | Costs of the vocational rehabilitation program were estimated at €1,426, of  which approximately 20% included time and travel costs incurred by the patients. No significant differences were found in other health care consumption, productivity, or QALYs. The program costs were outweighed by total savings on other health care and non-health care costs, but not significantly. | 73 |
| Van Meijeren-Pont et al. (2021)  Netherlands | To estimate the 1-year societal costs from the start of the rehabilitation in stroke patients treated in a medical specialist rehabilitation facility in The Netherlands, and to evaluate health changes in terms of utility over that year. | - adult/older adult - stroke | - inpatient rehabilitation - rehabilitation | No | - prospective observational cohort study - cost description - cost-benefit analysis - Perspective: societal (service costs, service user out-of-pocket costs, informal care, paid home-care, & productivity loss) | The mean 1-year costs for inpatient and outpatient rehabilitation were US$70,601 and US$27,473, respectively. For both inpatients and outpatients, rehabilitation was the biggest contributor, yet to a larger extent in inpatients than in outpatients. Productivity loss and informal care were other large contributors to the costs for both inpatients and outpatients. Between baseline and 6 months, and baseline and 12 months, utility improved significantly for inpatients. | 69 |
| Vincent & Vincent (2008)  United States | To characterize and compare short-term inpatient rehabilitation clinical and economical outcomes in cardiopulmonary patients referred to inpatient rehabilitation facilities and skilled nursing facilities environments. | - adult/older adult - cardiovascular problems - pulmonary disease | - activities of daily living - assistive technology - group intervention - inpatient rehabilitation - therapeutic exercise | No | - retrospective comparative study - cost description - Perspective: service costs | More patients achieved functional independence (significant), had shorter length of stay (significant), and had a higher rate of homebound discharge (significant) in the inpatient rehabilitation facility (IRF) than in the skilled nursing facility. These differences are associated with a higher cost of care (significant) in the IRF. | 49 |
| Visser et al. (2023)  Netherlands | To assess the cost-effectiveness of a cardiac rehabilitation (CR) program (OPTICARE XL) specifically designed for cardiac patients with obesity versus standard CR. | - adult/older adult - cardiovascular problems | - health problem prevention - health promotion - rehabilitation | No | - randomized controlled trial - cost effectiveness analysis - Perspective: societal (service costs, service user non-health care costs, & caregiver costs) | No differences between OPTICARE XL cardiac rehabilitation (CR) and standard CR in health effects and costs were revealed. | 79.8 |
| Von Koch et al. (2001)  Sweden | To evaluate early supported discharge and continued rehabilitation at home after stroke, at a minimum of 6 months after the intervention, in terms of patient outcome, resource use and health care cost. | - adult/older adult - stroke | - activities of daily living - home-based occupational therapy | No | - randomized controlled trial - cost analysis - Perspective: service costs | A significant difference in inpatient hospital care, initial and recurrent, was observed (higher in control group). Further significant differences were that the control group registered more outpatient visits to hospital OTs, private physical therapists and day-hospital attendance, while the intervention group registered more visits to nurses in primary care and home rehabilitation. Other differences in outcomes or resource utilization were nonsignificant. | 38 |
| Vos-Vromans et al. (2017)  Netherlands | To report the cost-effectiveness and cost-utility from a societal perspective comparing Multidisciplinary rehabilitation treatment (MRT) and cognitive behavioral therapy (CBT) in terms of reduction in fatigue and gain in health-related quality of life and gains in QALYs. | - adult - chronic illness | occupation-focused | No | - randomized controlled trial - cost effectiveness analysis - cost utility analysis - Perspective: societal (service costs, service user & caregiver costs, productivity losses) | Multidisciplinary rehabilitation treatment (MRT) was significantly more effective in reducing fatigue at 52 weeks. The mean difference in QALY between the treatments was not significant. The total societal costs were significantly higher for patients allocated to MRT. MRT has a high probability of being the most cost effective, using fatigue as the primary outcome. The incremental cost-effectiveness ratio is €856 per unit of the Checklist Individual Strength fatigue subscale. The results of the cost-utility analysis, using the QALY, indicate that the cognitive behavioral therapy had a higher likelihood of being more cost effective. | 100 |
| Wales et al. (2018)  Australia | To compare the cost effectiveness of two occupational therapy-led discharge planning interventions from the HOME trial. | - older adult - not specified | - home assessment - home-based occupational therapy | Yes | - randomized controlled trial - cost effectiveness analysis - Perspective: service costs | The cost of the enhanced OT discharge planning program (HOME) was nearly twice as high as that of the in-hospital consultation. However, a higher proportion of patients showed improvement in activities of daily living in the enhanced program with an incremental cost-effectiveness ratio of AUD$61,906.00 per person with clinically meaningful improvement. At a willingness to pay threshold of AUD$50,000.00 per additional patient with clinically significant improvement in the Nottingham Extended Activities of Daily Living, there is a 40% probability that the enhanced program is cost effective. | 93 |
| Wijnen et al. (2019)  Netherlands | To evaluate whether a new rehabilitation service for multitrauma patients (FT), when compared to conventional trauma rehabilitation care (CAU), would be preferable in terms of costs, effects and utilities from a societal perspective. | - adult - orthopedic | - activities of daily living - rehabilitation | No | - prospective observational cohort study - cost effectiveness analysis - cost utility analysis - Perspective: societal (service costs, service user & caregiver informal care costs, paid domestic help, out-of-pocket costs, aid, in-home modifications, productivity losses) | The bootstrapped incremental effectiveness on the Functional Independence Measure (FIM) was 3.7 points in favor of the Fast Track (FT) group and the incremental bootstrapped (extra) costs were €19,034, resulting in an incremental cost-effectiveness ratio (ICER) for cost per improvement on the FIM of €5,177. There is a 73% chance that the FT rehabilitation is more cost effective, given a border value of €20,000; however, in the absence of a willingness-to-pay threshold for such a clinical measure, no statements regarding its cost-effectiveness can be made. For cost per QALY, there was only a 4% chance that the FT rehabilitation program is more cost-effective than the Care-as-usual rehabilitation, suggesting that FT is not cost-effective. | 91 |
| Williamson et al. (2023)  United Kingdom | The aim of this study was to document individual-level community health and long-term care service usage for an exemplar cohort of people with severe obesity. | - adult/older adult - obesity | - activities of daily living - home adaptation - home-based occupational therapy | No | - description - cost analysis - Perspective: service costs | 22 different cross-sector community health and long-term care (LTC) services were used, including community equipment service, district nursing, OT, and LTC. 24 (96%) participants used 3 or more services, with longest care episode lasting over 14 years. The total annual service costs incurred by participants varied from £2053-82 792 (mean £26 594), with greatest costs being for LTC. Individual costs for equipment (currently provided) and home adaptations (ever provided) ranged widely, from zero-£35 946. Total mean annual costs increased by ascending BMI category, up to BMI 70 kg/m2. | 62.6 |
| Xie et al. (2021)  Canada | To compare the time on task and costs between MIH (Mobile Integrated Health Care) and matched ambulance services from a public health care payer’s perspective using real-world data routinely collected by the Emergency Medical Services system. | - adult/older adult - not specified | - fall prevention - hospital admission prevention - home-based occupational therapy | No | - retrospective chart review - randomized controlled trial - cost effectiveness analysis - Perspective: service costs | On average, the Mobile Integrated Health Care Team (MIH) team spent approximately 10-15 minutes longer on scene but reduced Emergency Department transport by 45-50%, each of which took approximately 40 minutes of ambulance service time. The cost was substantially higher for regular ambulance response than for MIH, with the difference ranging from CDN$163,000-$180,000 in the base-case and sensitivity analyses. MIH was associated with savings of approximately 60% of the total cost compared with matched ambulances. | 100 |
| Yoshida et al. (2019)  Japan | This study aimed to examine the effect of adding the adjusting the challenge-skill balance to the rehabilitation process on the subjective quality of life as well as on the cost-effectiveness. | - adult/older adult - brain injury, spinal cord injury, cerebrovascular accident | - inpatient rehabilitation - occupation-focused - rehabilitation | Yes | - randomized controlled trial - cost effectiveness analysis - Perspective: service costs | Significant differences were found in the Ikigai-9 score and the EQ-5D-5L during the entire period as well as in the pre-post-assessment. No significant differences were found for the Flow State Scale for Occupational Tasks or the Functional Independence Measure. The cost of treatment in the experimental group was US$1710.86, compared to US$1659.46 in the control group. This produced quality-adjusted life-year of 0.173 and 0.164 in the experimental and control groups, respectively. The incremental cost-effectiveness ratio was US$5518.38 per QALY. | 52.5 |
| Yu et al. (2004)  Hong Kong | To evaluate the long-term effect of a cardiac rehabilitation and prevention program on quality of life and its cost effectiveness. | - adult/older adult - cardiovascular problems | - health problem prevention - lifestyle intervention - rehabilitation | No | - randomized controlled trial - cost effectiveness analysis - Perspective: service costs | The direct health care expenses in the cardiac rehabilitation and prevention program (CRPP) and control groups were US$15,292 and US$15,707 per patient, respectively. Therefore, the cost utility calculated was $$640 saved per QALY gained. Savings attributable to CRPP were primarily explained by the significantly lower rate and cost of subsequent percutaneous coronary intervention (PCI). | 73 |
| Zeidler et al. (2008)  Germany | To examine the costs of inpatient and outpatient rehabilitation for musculoskeletal disorders from the perspective of a major statutory health insurance fund in Germany. | - adult/older adult - musculoskeletal condition | - inpatient rehabilitation - rehabilitation | No | - retrospective chart review - cost analysis - Perspective: service costs | After a preceding hospital stay, inpatient and outpatient rehabilitation results in mean costs of €2047 and €1111, respectively. If the rehabilitation was not preceded by a directly related hospital treatment, mean costs for inpatient (outpatient) rehabilitation were €2067 (€1310). No systematic differences could be found between inpatient and outpatient rehabilitation evaluating costs for hospital treatment,  drugs or physical therapy in the year preceding and the year directly following the rehabilitation. | 81 |
| Zhao et al. (2009)  China | The aim of this primary research is to explore the service and treatment options of Traditional Chinese Medicine and western medicine therapies for patients receiving rehabilitative services and their satisfaction with these services in China’s Shandong Province. Indicators of patient satisfaction with services provided, patient participation in treatment decisions, and satisfaction with their payer are also explored. | - adult/older adult - not specified | - activities of daily living - return-to-work intervention | No | - description - cost description - Perspective: societal (service costs, service user contribution to costs) | The most frequently ordered traditional Chinese Medicine therapies were acupuncture and massage therapy. The most commonly prescribed Western Medicine rehabilitative treatments were physical therapy and OT. On average, the cost for rehabilitative therapies was about 29 Yuan (US$4.20) per visit with an average of 6 visits per week. This cost is still high relative to patients’ reported monthly income. The monthly cost for rehabilitative therapies would be approximately 696 Yuan (US$99); just over 19% of the patients in this study had a monthly income of less than 2000 Yuan (US$286). | 32 |
| Zingmark et al. (2016)  Sweden | To evaluate three occupational therapy interventions, focused on supporting continued engagement in occupation among older people, to determine which intervention was most cost effective, evaluated as the incremental cost/quality adjusted life year gained, in relation to a control group. | - older adult - not specified | occupation-focused | Yes | - randomized controlled trial - cost effectiveness analysis - Perspective: societal (however, only service costs included) | The individual intervention (IG) was not cost effective. The activity group (AG) appeared to be potentially cost effective at 3 months, but not at 12 months. The discussion group (DG) appeared to be cost-effective at 3 months and potentially cost effective at 12 months. When the QALYs gained at 3 months were adjusted for general health at baseline, the AG was more effective than the DG. In both AG and DG, the difference was significant in relation to the CG. Considering the incremental total cost for the AG in relation to DG, the incremental cost-effectiveness ratio (ICER) was €197,000/QALY gained. However, the difference in total cost for the AG in relation to the CG was not significant. If the estimate of ICER was based on the difference in intervention cost only, it was €64,000/QALY gained for the AG. | 92 |
| Zingmark et al. (2017)  Sweden | To evaluate the cost effectiveness of an intervention implemented to minimize bathing disability for older people with bathing disability. | - older adult - not specified | - activities of daily living - home-based occupational therapy | Yes | - retrospective comparative study - cost effectiveness analysis - Perspective: societal (service costs, caregiver time, special accommodation) | Over the full follow-up period, the intervention (minimizing bathing disability) resulted in QALY gains and reduced societal cost. After 8 years, the intervention resulted in 0.052 QALYs gained and reduced societal costs by €2410 per person. In comparison to the intervention cost, the intervention effect was a more important factor for the magnitude of QALY gains and long-term societal costs. The intervention cost had only minor impact on societal costs. | 86 |
| Zorowitz et al. (2009)  United States | To examine mortality, costs, and rehabilitation use in patients with stroke and stroke-related hemiparesis during a 4-year period following stroke onset. | - older adult - stroke | rehabilitation | No | - retrospective chart review - cost description - Perspective: service costs | The average Medicare cost per patient over the 4-year period was US$77,143 for the hemiparesis cohort and US$53,319 for the nonhemiparesis cohort. A significantly higher proportion of patients in the hemiparesis cohort received rehabilitation than in the nonhemiparesis cohort. Among patients who received rehabilitation, costs were significantly higher for the hemiparesis cohort than for the nonhemiparesis cohort in the first year. While most rehabilitation costs for the hemiparesis cohort were incurred in the hospital inpatient setting in the first year, the cost burden shifted to skilled nursing facilities and home health agencies in the following 3 years. | 93 |

*Throughout this study findings column, for the sake of brevity, « OT » is used to replace *occupational therapy* and *occupational therapist*

** The occupational therapy role in the intervention being evaluated was clearly identified.

**References**

- Ah-Soune, M. F., & De Vignerte, B. (2000). Interest and costs of home reinsertion after a stay in geriatric rehabilitation unit. *Revue de gériatrie*, *25*(7), 471-476.
- Allen, L., John-Baptiste, A., Meyer, M., Richardson, M., Speechley, M., Ure, D., Markle-Reid, M., & Teasell, R. (2019). Assessing the impact of a home-based stroke rehabilitation programme: A cost-effectiveness study. *Disability & Rehabilitation*, *41*(17), 2060-2065. <https://doi.org/https://doi.org/10.1080/09638288.2018.1459879>
- Anderson, C., Mhurchu, C. N., Rubenach, S., Clark, M., Spencer, C., & Winsor, A. (2000). Home or hospital for stroke rehabilitation? Results of a randomized controlled trial : II: cost minimization analysis at 6 months. *Stroke*, *31*(5), 1032-1037. <https://doi.org/10.1161/01.str.31.5.1032>
- Andersson, A., Levin, L. K., Öberg, B., & Månsson, L. (2002). Health care and social welfare costs in home-based and hospital-based rehabilitation after stroke. *Scandinavian Journal of Caring Sciences*, *16*(4), 386-392. <https://doi.org/https://doi.org/10.1046/j.1471-6712.2002.00115.x>
- Angelo, M., Vass, R., Flores Vazquez, I., Pierre, D., Del Vecchio, T., & Souder, E. (2021). Association between outpatient rehabilitation therapy and total cost of care for a frail elderly population in a Medicare Accountable Care Organization. *Population Health Management*, *24*(1), 110-115. <https://doi.org/http://doi.org/10.1089/pop.2019.0223>
- Angerová, Y., Maršálek, P., Chmelová, I., Gueye, T., Barták, M., Uherek, S., Bříza, J., & Rogalewicz, V. (2021). Cost analysis of early rehabilitation after stroke in comprehensive cerebrovascular centres in the Czech Republic. *Central European Journal of Public Health*, *29*(2), 153-158. <https://doi.org/10.21101/cejph.a6111>
- Angerová, Y., Maršálek, P., Chmelová, I., Gueye, T., Uherek, S., Bříza, J., Barták, M., & Rogalewicz, V. (2020). Cost and cost-effectiveness of early inpatient rehabilitation after stroke varies with initial disability: the Czech Republic perspective. *International Journal of Rehabilitation Research*, *43*(4), 376-382. <https://doi.org/10.1097/MRR.0000000000000440>
- Archongka, Y., Manimmanakorn, N., Kuptniratsaikul, V., & Yeeheng, P. (2008). Unit cost of stroke rehabilitation. *Journal of the Medical Association of Thailand*, *91*(8), 1257-1262.
- Bendixen, R. M., Levy, C. E., Olive, E. S., Kobb, R. F., & Mann, W. C. (2009). Cost effectiveness of a telerehabilitation program to support chronically ill and disabled elders in their homes. *Telemedicine and E-Health*, *15*(1), 31-38. <https://doi.org/https://doi.org/10.1089/tmj.2008.0046>
- Björkdahl, A., & Sunnerhagen, K. S. (2007). Process skill rather than motor skill seems to be a predictor of costs for rehabilitation after a stroke in working age: A longitudinal study with a 1 year follow up post discharge. *Health Services Research*, *7*(1), 209. <https://doi.org/https://doi.org/10.1186/1472-6963-7-209>
- Breysse, J., Dixon, S., Wilson, J., & Szanton, S. L. (2022). Aging gracefully in place: An evaluation of the capability of the CAPABLE approach. *Journal of Applied Gerontology*, *41*(3), 718-728. <https://doi.org/10.1177/07334648211042606>
- Brusco, N. K., Voogt, A., Nott, M., Callaway, L., Mansoubi, M., & Layton, N. (2022). Meeting unmet needs for stroke rehabilitation in rural public health: Explorative economic evaluation of upper limb robotics-based technologies through a capabilities lens. *Societies*, *12*(143). <https://doi.org/https://doi.org/10.3390/soc12050143>
- Brusco, N. K., Watts, J. J., Shields, N., & Taylor, N. (2015). Is cost effectiveness sustained after weekend inpatient rehabilitation? 12 month follow up from a randomized controlled trial. *BMC Health Services Research*, *15*(1), 1-15. <https://doi.org/https://doi.org/10.1186/s12913-015-0822-3>
- Brusco, N. K., Watts, J. J., Shields, N., & Taylor, N. F. (2014). Are weekend inpatient rehabilitation services value for money? An economic evaluation alongside a randomized controlled trial with a 30 day follow up. *BMC Medicine*, *12*, 89. <https://doi.org/https://doi.org/10.1186/1741-7015-12-89>
- Burns, D. K., Wilson, E. C. F., Browne, P., Olive, S., Clark, A., Galey, P., Dix, E., Woodhouse, H., Robinson, S., & Wilson, A. (2016). The cost effectiveness of maintenance schedules following pulmonary rehabilitation in patients with chronic obstructive pulmonary disease: An economic evaluation alongside a randomised controlled trial. *Applied Health Economics and Health Policy*, *14*(1), 105-115. <https://doi.org/https://doi.org/10.1007/s40258-015-0199-9>
- Campbell, A. J., Robertson, M. C., La Grow, S. J., Kerse, N. M., Sanderson, G. F., Jacobs, R. J., Sharp, D. M., & Hale, L. A. (2005). Randomised controlled trial of prevention of falls in people aged ≥75 with severe visual impairment: The VIP trial. *British Medical Journal*, *331*(7520), 817-820. <https://doi.org/10.1136/bmj.38601.447731.55>
- Carande-Kulis, V., Stevens, J. A., Florence, C. S., Beattie, B. L., & Arias, I. (2015). A cost–benefit analysis of three older adult fall prevention interventions. *Journal of Safety Research*, *52*, 65-70. <https://doi.org/https://doi.org/10.1016/j.jsr.2014.12.007>
- Carlill, G., Gash, E., & Hawkins, G. (2002). Preventing unnecessary hospital admissions: An occupational therapy and social work service in an accident and emergency department. *British Journal of Occupational Therapy*, *65*(10), 440-445. <https://doi.org/https://doi.org/10.1177/030802260206501002>
- Chen, Y.-C., Yeh, Y.-J., Wang, C.-Y., Lin, H.-F., Lin, C.-H., Hsien, H.-H., Hung, K.-W., Wang, J.-D., & Shi, H.-Y. (2022). Cost utility analysis of multidisciplinary postacute care for stroke: A prospective six-hospital cohort study. *Frontiers in Cardiovascular Medicine*, *30*(9), 826898. <https://doi.org/10.3389/fcvm.2022.826898>
- Chew, D. S., Li, Y. C., Zeitouni, M., Whellan, D. J., Kitzman, D., Mentz, R. J., Duncan, P., Pastva, A. M., Reeves, G. R., Nelson, M. B., Chen, H.-C., & Reed, S. D. (2022). Economic outcomes of rehabilitation therapy in older patients with acute heart failure in the REHAB-HF trial: A secondary analysis of a randomized clinical trial. *JAMA Cardiology*, *7*(2), 140-148. <https://doi.org/https://doi.org/10.1001/jamacardio.2021.4836>
- Chou, H.-Y., Tsai, Y.-W., Ma, S.-C., Ma, S.-M., & Shih, C.-L. (2023). Efficacy and cost over 12 hospitalization weeks of postacute care for stroke. *International Journal of Environmental Research and Public Health*, *20*(2), 1419. <https://doi.org/10.3390/ijerph20021419>
- Clare, L., Kudlicka, A., Collins, R., Evans, S. B., Pool, J., Henderson, C., Knapp, M., Litherland, R., Oyebode, J., & Woods, R. (2023). Implementing a home-based personalised cognitive rehabilitation intervention for people with mild-to-moderate dementia: GREAT into Practice. *BMC Geriatrics*, *23*(1), 93. <https://doi.org/10.1186/s12877-022-03705-0>
- Clark, F., Jackson, J., Carlson, M., Chou, C. P., Cherry, B. J., Jordan-Marsh, M., Knight, B. G., Mandel, D., Blanchard, J., Granger, D. A., Wilcox, R. R., Lai, M. Y., White, B., Hay, J., Lamb, C., Marterella, A., & Azen, S. P. (2012). Effectiveness of a lifestyle intervention in promoting the well-being of independently living older adults: Results of the Well Elderly 2 randomised controlled trial. *Journal of Epidemiology & Community Health*, *66*(9), 782-790. <https://doi.org/10.1136/jech.2009.099754>
- Clarke, C. E., Patel, S., Ives, N., Rick, C. E., Woolley, R., Wheatley, K., Walker, M. F., Zhu, S., Kandiyali, R., Yao, G., & Sackley, C. M. (2016). Clinical effectiveness and cost-effectiveness of physiotherapy and occupational therapy versus no therapy in mild to moderate Parkinson’s disease: A large pragmatic randomised controlled trial (PD REHAB). *Health Technology Assessment*, *20*(63), 1-96. <https://doi.org/https://doi.org/10.3310/hta20630>
- Cook, S., & Howe, A. (2003). Engaging people with enduring psychotic conditions in primary mental health care and occupational therapy. *British Journal of Occupational Therapy*, *66*(6), 236-246. <https://doi.org/https://doi.org/10.1177/030802260306600602>
- Cooney, M. T., & Carroll, A. (2016). Cost effectiveness of inpatient rehabilitation in patients with brain injury. *Clinical Medicine*, *16*(2), 109-113. <https://doi.org/10.7861/clinmedicine.16-2-109>
- Cunningham, J. L., Rome, J. D., Kerkvliet, J. L., & Townsend, C. O. (2009). Reduction in medication costs for patients with chronic nonmalignant pain completing a pain rehabilitation program: A prospective analysis of admission, discharge, and 6-month follow-up medication costs. *Pain Medicine*, *10*(5), 787-796. <https://doi.org/https://doi.org/10.1111/j.1526-4637.2009.00582.x>
- Curtis, L., & Beecham, J. (2018). A survey of local authorities and home improvement agencies: Identifying the hidden costs of providing a home adaptations service. *British Journal of Occupational Therapy*, *81*(11), 633-640. <https://doi.org/10.1177/0308022618771534>
- Duru, O. K., Ettner, S. L., Vassar, S. D., Chodosh, J., & Vickrey, B. G. (2009). Cost evaluation of a coordinated care management intervention for dementia. *American Journal of Managed Care*, *15*(8), 521-528.
- Edelstein, J., Walker, R., Middleton, A., Reistetter, T., Williams Gary, K., & Reynolds, S. (2022). Higher frequency of acute occupational therapy services is associated with reduced hospital readmissions. *American Journal of Occupational Therapy*, *76*(1), 7601180090. <https://doi.org/https://doi.org/10.5014/ajot.2022.048678>
- Eklund, K., Stålnacke, B.-M., Stenberg, G., Enthoven, P., Gerdle, B., & Sahlén, K.-G. (2021). A cost-utility analysis of multimodal pain rehabilitation in primary healthcare. *Scandinavian Journal of Pain*, *21*(1), 48-58. <https://doi.org/https://doi.org/10.1515/sjpain-2020-0050>
- Ellis, A., Trappes-Lomax, T., Fox, M., Taylor, R., Power, M. L., Stead, J., & Bainbridge, I. F. (2006). Buying Time II: An economic evaluation of a joint NHS/Social Services residential rehabilitation unit for older people on discharge from hospital. *Health & Social Care in the Community*, *14*(2), 95-106. <https://doi.org/https://doi.org/10.1111/j.1365-2524.2006.00597.x>
- Evans, J. R., Benore, E., & Banez, G. A. (2016). The cost-effectiveness of intensive interdisciplinary pediatric chronic pain rehabilitation. *Journal of Pediatric Psychology*, *41*(8), 849-856. <https://doi.org/10.1093/jpepsy/jsv100>
- Everink, I. H. J., van Haastregt, J. C. M., Evers, S. M. A. A., Kempen, G. I. J. M., & Schols, J. M. G. A. (2018). An economic evaluation of an integrated care pathway in geriatric rehabilitation for older patients with complex health problems. *PLoS One*, *13*(2), e0191851. <https://doi.org/https://doi.org/10.1371/journal.pone.0191851>
- Firzah Abdul Aziz, A., Azlin Mohd Nordin, N., Nur Amrizal, M., Saperi, S., & Syed Mohamed, A. (2020). The integrated care pathway for managing post stroke patients (iCaPPS©) in public primary care Healthcentres in Malaysia: Impact on quality adjusted life years
- (QALYs) and cost effectiveness analysis. *BMC Geriatrics*, *20*(1), 70. <https://doi.org/https://doi.org/10.1186/s12877-020-1453-z>
- Flood, C., Mugford, M., Stewart, S., Harvey, I., Poland, F., & Lloyd-Smith, W. (2005). Occupational therapy compared with social work assessment for older people. An economic evaluation alongside the CAMELOT randomised controlled trial. *Age and Ageing*, *34*(1), 47-52. <https://doi.org/10.1093/ageing/afh232>
- Gage, H., Kaye, J., Owen, C., Trend, P., & Wade, D. (2006). Evaluating rehabilitation using cost-consequences analysis: An example in Parkinson’s disease. *Clinical Rehabilitation*, *30*(3), 232-238. <https://doi.org/https://doi.org/10.1191/0269215506cr936oa>
- Gieser, D. K., Williams, R. T., O'Connell, W., Pasquale, L. R., Rosenthal, B. P., Walt, J. G., Katz, L. M., Siegartel, L. R., Wang, L.-H., Rosenblatt, L. C., Stern, L. S., & Doyle, J. J. (2006). Costs and utilization of end-stage glaucoma patients receiving visual rehabilitation care: A US multisite retrospective study. *Journal of Glaucoma*, *15*(5), 419-425. <https://doi.org/10.1097/01.ijg.0000212250.95078.6f>
- Gillespie, P., Hobbins, A., O'Toole, L., Connolly, D., Boland, F., & Smith, S. M. (2022). Cost-effectiveness of an occupational therapy-led self-management support programme for multimorbidity in primary care. *Family Practice*, *39*, 826-833. <https://doi.org/10.1093/fampra/cmac006>
- Gitlin, L. N., Hodgson, N., Jutkowitz, E., & Pizzi, L. (2010). The cost-effectivness of a nonpharmacologic intervention for individuals with dementia and family caregivers: The tailored activity program. *American Journal of Geriatric Psychiatry*, *18*(6), 510-519. <https://doi.org/10.1097/JGP.0b013e3181c37d13>
- Godwin, K. M., Wasserman, J., & Ostwald, S. K. (2011). Cost associated with stroke: Outpatient rehabilitative services and medication. *Topics in Stroke Rehabilitation*, *18*, 676-684. <https://doi.org/10.1310/tsr18s01-676>
- Gospodarevskaya, E., Carter, R., Imms, C., Yee Chu, E. M., Nicola-Richmond, K. M., Gribble, N., Froude, E., Guinea, S., Sheppard, L., Iezzi, A., & Chen, G. (2019). Economic evaluation of simulated and traditional clinical placements in occupational therapy education. *Australian Occupational Therapy Journal*, *66*(3), 369-379. <https://doi.org/10.1111/1440-1630.12563>
- Graff, M. J., Adang, E. M., Vernooij-Dassen, M. J., Dekker, J., Jonsson, L., Thijssen, M., Hoefnagels, W. H., & Rikkert, M. G. (2008). Community occupational therapy for older patients with dementia and their care givers: Cost effectiveness study. *British Medical Journal*, *336*(7636), 134-138. <https://doi.org/10.1136/bmj.39408.481898.BE>
- Griffiths, T., Phillips, C., Davies, S., Burr, M., & Campbell, I. (2001). Cost effectiveness of an outpatient multidisciplinary pulmonary rehabilitation programme. *Thorax*, *56*(10), 779-784. <https://doi.org/10.1136/thorax.56.10.779>
- Grimmer, K., & May, E. (2001). Cost drivers of public hospital occupational therapy outpatient care. *Australian Occupational Therapy Journal*, *48*(4), 150-156. <https://doi.org/https://doi.org/10.1046/j.1440-1630.2001.00245.x>
- Haines, T. P., Bowles, K.-A., Mitchell, D., O'Brien, L., Markham, D., Plumb, S., May, K., Philip, K., Haas, R., Sarkies, M. N., Ghaly, M., Shackell, M., Chiu, T., McPhail, S., McDermott, F., & Skinner, E. H. (2017). Impact of disinvestment from weekend allied health services across acute medical and surgical wards: 2 stepped-wedge cluster randomised controlled trials. *PLoS Medicine*, *14*(10), e1002412. <https://doi.org/10.1371/journal.pmed.1002412>
- Harper, K. J., McAuliffe, K., Williamson, M., Jacques, A., Sainsbury, K., & Edwards, D. (2024). An occupational therapy delirium pathway reduces hospital re-presentations in older adults with delirium: A before and after observational study. *British Journal of Occupational Therapy*, *87*(2), 79-88. <https://doi.org/https://doi-org.acces.bibl.ulaval.ca/10.1177/03080226231197010>
- Hay, J., LaBree, L., Luo, R., Clark, F., Carlson, M., Mandel, D., Zemke, R., Jackson, J., & Azen, S. P. (2002). Cost-effectiveness of preventive occupational therapy for independent-living older adults. *Journal of the American Geriatrics Society*, *50*(8), 1381-1388. <https://doi.org/10.1046/j.1532-5415.2002.50359.x>
- Hendriks, M. R. C., Evers, S. M. A. A., Bleijlevens, M. H. C., van Haastregt, J. C. M., Crebolder, H. F. J. M., & van Eijk, J. T. M. (2008). Cost-effectiveness of a multidisciplinary fall prevention program in community-dwelling elderly people: A randomized controlled trial (ISRCTN 64716113). *International Journal of Technology Assessment in Health Care*, *24*(2), 193-202. <https://doi.org/10.1017/S0266462308080276>
- Hutchinson, C., Berndt, A., Cleland, J., Gilbert-Hunt, S., George, S., & Ratcliffe, J. (2020). Using social return on investment analysis to calculate the social impact of modified vehicles for people with disability. *Australian Occupational Therapy Journal*, *67*(3), 250-259. <https://doi.org/https://doi.org/10.1111/1440-1630.12648>
- Hwang, R., Morris, N. R., Mandrusiak, A., Bruning, J., Peters, R. D., Korczyk, D., & Russell, T. (2018). Cost-utility analysis of home-based telerehabilitation compared with centre-based rehabilitation in patients with heart failure. *Heart, Lung and Circulation*, *28*(12), 1795-1803. <https://doi.org/10.1016/j.hlc.2018.11.010>
- Ikiugu, M. N., & Anderson, L. (2007). Cost effectiveness of the instrumentalism in occupational therapy (IOT) conceptual model as a guide for intervention with adolescents with emotional and behavioral disorders (EBD). *International Journal of Behavioral and Consultation Therapy*, *3*(1), 53-76. <https://doi.org/http://dx.doi.org/10.1037/h0100175>
- Irvine, L., Conroy, S. P., Sach, T., Gladman, J. R., Harwood, R. H., Kendrick, D., Coupland, C., Drummond, A., Barton, G., & Masud, T. (2010). Cost-effectiveness of a day hospital falls prevention programme for screened community-dwelling older people at high risk of falls. *Age and Ageing*, *39*(6), 710-716. <https://doi.org/10.1093/ageing/afq108>
- Isarunuwatchai, W., Perdrizet, J., Markle-Reid, M., & Hoch, J. S. (2017). Cost-effectiveness analysis of a multifactorial fall prevention in older home care clients at risk for falling. *BMC Geriatrics*, *17*, 199. <https://doi.org/10.1186/s12877-017-0599-9>
- Jenkyn, K. B., Hoch, J. S., & Speechley, M. (2012). How much are we willing to pay to prevent a fall? Cost-effectiveness of multifactorial falls prevention program for community-dwelling older adults. *Canadian Journal on Aging*, *31*(2), 121-137. <https://doi.org/10.1017/S0714980812000074>
- Jutkowitz, E., Gitlin, L. N., Pizzi, L. T., Lee, E., & Dennis, M. P. (2012). Cost effectiveness of a home-based intervention that helps functionally vulnerable older adults age in place at home. *Journal of Aging Research*, *2012*, 680265. <https://doi.org/10.1155/2012/680265>
- Kehusmaa, S., Autti-Rämö, I., Valaste, M., Hinkka, K., & Rissanen, P. (2010). Economic evaluation of a geriatric rehabilitation programme: A randomized controlled trial. *Journal of Rehabilitation Medicine*, *42*(10), 949-955. <https://doi.org/https://doi.org/10.2340/16501977-0623>
- Kennedy, N., Stokes, E., O'Shea, E., Murphy, T. E., Bresnihan, B., & FitzGerald, O. (2007). Inpatient and outpatient rehabilitation for patients with rheumatoid arthritis: A clinical and economic assessment. *Journal of Medical Economics*, *10*(4), 515-528. <https://doi.org/https://doi.org/10.3111/13696990701725850>
- Khiaocharoen, O., Pannarunothai, S., Riewpaiboon, W., Ingsrisawang, L., & Teerawattananon, Y. (2012). Economic evaluation of rehabilitation services for inpatients with stroke in Thailand: A prospective cohort study. *Value in Health Regional Issues*, *1*(1), 29-35. <https://doi.org/https://doi.org/10.1016/j.vhri.2012.03.021>
- Kiekens, C., Van Rie, K., Peers, K., & Lysens, R. (2011). Cost of rehabilitation care in traumatic and nontraumatic spinal cord injury in a European context. *Topics in Spinal Cord Injury Rehabilitation*, *16*(4), 43-52. <https://doi.org/https://doi.org/10.1310/sci1604-43>
- Killaspy, H., Marston, L., Green, N., Harrison, I., Lean, M., Holloway, F., Craig, T., Leavey, G., Arbuthnott, M., Koeser, L., McCrone, P., Omar, R. Z., & King, M. (2016). Clinical outcomes and costs for people with complex psychosis: A naturalistic prospective cohort study of mental health rehabilitation service users in England. *BMC Psychiatry*, *16*(1), 95. <https://doi.org/https://doi.org/10.1186/s12888-016-0797-6>
- Kim, S. W., Jeon, H. R., Youk, T., & Kim, J. (2018). Cost of rehabilitation treatment of patients with cerebral palsy in Korea. *Annals of Rehabilitation Medicine*, *42*(5), 722-729. <https://doi.org/10.5535/arm.2018.42.5.722>
- Lahtinen, A., Leppilahti, J., Vähänikkilä, H., Harmainen, S., Koistinen, P., Rissanen, P., & Jalovaara, P. (2017). Costs after hip fracture in independently living patients: a randomised comparison of three rehabilitation modalities. *Clinical Rehabilitation*, *31*(5), 672-685. <https://doi.org/10.1177/0269215516651480>
- Lambert, R. A., Lorgelly, P., Harvey, I., & Poland, F. (2010). Cost-effectiveness analysis of an occupational therapy-led lifestyle approach and routine general practitioner's care for panic disorder. *Social Psychiatry & Psychiatric Epidemiology*, *45*(7), 741-750. <https://doi.org/10.1007/s00127-009-0114-5>
- Larsen, K., Hansen, T. B., Thomsen, P. B., Christiansen, T., & Søballe, K. (2009). Cost-effectiveness of accelerated perioperative care and rehabilitation after total hip and knee arthroplasty. *Journal of Bone and Joint Surgery*, *91*(4), 761-772. <https://doi.org/http://doi.org/10.2106/JBJS.G.01472>
- Lewin, G., Allan, J., Patterson, C., Knuiman, M., Boldy, D., & Hendrie, D. (2014). A comparison of the home-care and healthcare service use and costs of older Australians randomised to receive a restorative or a conventional home-care service. *Health & Social Care in the Community*, *22*(3), 328-336. <https://doi.org/10.1111/hsc.12092>
- Li, H. J., Chen, C. Y., Tsai, C. H., Kuo, C. C., Chen, K. H., Chen, K. H., & Li, Y. C. (2019). Utilization and medical costs of outpatient rehabilitation among children with autism spectrum conditions in Taiwan. *BMC Health Services Research*, *19*, 354. <https://doi.org/https://doi.org/10.1186/s12913-019-4193-z>
- Li, L. C., Maetzel, A., Davis, A. M., Lineker, S. C., Bombardier, C., & Coyte, P. C. (2006). Primary therapist model for patients referred for rheumatoid arthritis rehabilitation: A cost-effectiveness analysis. *Arthritis and Rheumatism*, *55*(3), 402-410. <https://doi.org/10.1002/art.21989>
- Loisel, P., Lemaire, J., Durand, M.-J., Champagne, F., Stock, S., Diallo, B., & Tremblay, C. (2002). Cost-benefit and cost-effectiveness analysis of a disability prevention model for back pain management: A six year follow up study. *Occupational & Environmental Medicine*, *59*(12), 807-815. <https://doi.org/10.1136/oem.59.12.807>
- Lord, R. K., Mayhew, C. R., Korupolu, R., Mantheiy, E. C., Friedman, M. A., Palmer, J. B., & Needham, D. M. (2013). ICU early physical rehabilitation programs: Financial modeling of cost savings. *Critical Care Medicine*, *41*(3), 717-724. <https://doi.org/https://doi.org/10.1097/CCM.0b013e3182711de2>
- Louw, Q., Twizeyemariya, A., Grimmer, K., & Leibbrandt, D. (2020). Estimating the costs and benefits of stroke rehabilitation in South Africa. *Journal of Evaluation in Clinical Practice*, *26*(4), 1181-1187. <https://doi.org/10.1111/jep.13287>
- Markle-Reid, M., Browne, G., Gafni, A., Roberts, J., Weir, R., Thabane, L., Miles, M. B., Vaitonis, V., Hecimovich, C., Baxter, P., & Henderson, S. (2010). The effects and costs of a multifactorial and interdisciplinary team approach to falls prevention for older home care clients ‘at risk’ for falling: A randomized controlled trial. *Canadian Journal of Aging*, *29*(1), 139-161. <https://doi.org/10.1017/S0714980809990377>
- Merkesdal, S., & Mau, W. (2005). Prediction of costs-of-illness in patients with low back pain undergoing orthopedic outpatient rehabilitation. *International Journal of Rehabilitation Research*, *28*(2), 119-126. <https://doi.org/http://doi.org/10.1097/00004356-200506000-00004>
- Miller, P., Gladman, J. R. F., Cunliffe, A. L., Husbands, S. L., Dewey, M. E., & Harwood, R. H. (2005). Economic analysis of an early discharge rehabilitation service for older people. *Age and Ageing*, *34*, 274-280. <https://doi.org/10.1093/ageing/afi058>
- Mortimer, D., Trevena-Peters, J., McKay, A., & Ponsford, J. (2019). Economic evaluation of activities of daily living retraining during posttraumatic amnesia for inpatient rehabilitation following severe traumatic brain injury. *Archives of Physical Medicine & Rehabilitation*, *100*(4), 648-655. <https://doi.org/10.1016/j.apmr.2018.08.184>
- Nagayama, H., Tomori, K., Ikeda, K., & Yamauchi, K. (2021). Medical costs and readmissions after intensive poststroke rehabilitation: Japanase claims data. *Journal of the American Medical Directors Association*, *22*(8), 1762-1766. <https://doi.org/10.1016/j.jamda.2020.12.015>
- Nagayama, H., Tomori, K., Ohno, K., Takahashi, K., Nagatani, R., Izumi, R., Moriwaki, K., & Yahauchi, K. (2017). Cost effectiveness of the occupation-based approach for subacute stroke patients: Result of a randomized controlled trial. *Topics in Stroke Rehabilitation*, *24*(5), 337-344. <https://doi.org/http://dx.doi.org/10.1080/10749357.2017.1289686>
- Nagayama, H., Tomori, K., Ohno, K., Takahashi, K., Ogahara, K., Sawada, T., Uezu, S., Nagatani, R., & Yamauchi, K. (2016). Effectiveness and cost-effectiveness of occupation-based occupational therapy using the aid for decision making in occupation choice (ADOC) for older residents: Pilot cluster randomized controlled trial. *PLoS One*, *11*(3), e0150374. <https://doi.org/https://doi.org/10.1371/journal.pone.0150374>
- Norrefalk, J.-R., Ekholm, K., Linder, J., Borg, K., & Ekholm, J. (2008). Evaluation of a multiprofessional rehabilitation programme for persistent musculoskeletal-related pain: Economic benefits of return to work. *Journal of Rehabilitation Medicine*, *40*(1), 15-22. <https://doi.org/https://doi.org/10.2340/16501977-0131>
- O'Connor, R. J., Beden, R., Pilling, A., & Chamberlain, M. A. (2011). What reductions in dependency costs result from treatment in an inpatient neurological rehabilitation unit for people with stroke? *Clinical Medicine (London, England)*, *11*(1), 40-43. <https://doi.org/10.7861/clinmedicine.11-1-40>
- Padwal, R. S., Wang, X., Sharma, A. M., & Dyer, D. (2012). The impact of severe obesity on post-acute rehabilitation efficiency, length of stay, and hospital costs. *Journal of Obesity*, *2012*, 972365. <https://doi.org/10.1155/2012/972365>
- Peeters, G. M. E. E., Heymans, M. W., de Vries, O. J., Bouter, L. M., Lips, P., & van Tulder, M. W. (2011). Multifactorial evaluation and treatment of persons with a high risk of recurrent falling was not cost-effective. *Osteoporosis International*, *22*(7), 2187-2196. <https://doi.org/10.1007/s00198-010-1438-4>
- Pergolotti, M., Lavery, J., Reeve, B. B., & Dusetzina, S. B. (2018). Therapy caps and variation in cost of outpatient occupational therapy by provider, insurance status, and geographic region. *American Journal of Occupational Therapy*, *72*(2), 1-9. <https://doi.org/10.5014/ajot.2018.023796>
- Pizzi, L. T., Jutkowitz, E., Prioli, K. M., Lu, E. Y., Babcock, Z., McAbee-Sevick, H., Wakefield, D. B., Robison, J., Molony, S., Piersol, C. V., Gitlin, L. N., & Fortinsky, R. H. (2022). Cost-benefit analysis of the COPE program for persons living with dementia: Toward a payment model. *Innovation in Aging*, *6*(1), 1-11. <https://doi.org/https://doi.org/10.1093/geroni/igab042>
- Pizzi, L. T., Prioli, K. M., Jutkowitz, E., Piersol, C. V., Lyketsos, C. G., Abersone, I., Marx, J. A., & Gitlin, L. N. (2023). Economic analysis of the Tailored Activity Program: A nonpharmacological approach to improve quality of life in people living with dementia and their caregivers. *Journal of Applied Gerontology*, *42*(7), 1433-1444. <https://doi.org/10.1177/07334648231158091>
- Pizzo, E., Wenborn, J., Burgess, J., Mundy, J., Orrell, M., King, M., Omar, R. Z., & Morris, S. (2022). Cost-utility analysis of community occupational therapy in dementia (COTiD-UK) versus usual care: Results from VALID, a multi-site randomised controlled trial in the UK. *PLoS One*, *17*(2), e0262828. <https://doi.org/https://doi.org/10.1371/journal.pone.0262828>
- Puolakka, K., Kautiainen, H., Möttönen, T., Hannonen, P., Pohjolainen, T., Korpela, M., & FIN‐RACo trial group. (2007). Cost of Finnish statutory inpatient rehabilitation and its impact on functional and work capacity of patients with early rheumatoid arthritis: Experience from the FIN‐RACo trial. *Scandinavian Journal of Rheumatology*, *36*(4), 270-277. <https://doi.org/https://doi.org/10.1080/03009740701286847>
- Radford, K., Phillips, J., Drummond, A., Sach, T., Walker, M., Tyerman, A., Haboubi, N., & Jones, T. (2013). Return to work after traumatic brain injury: Cohort comparison and economic evaluation. *Brain Injury*, *27*(5), 507-520. <https://doi.org/10.3109/02699052.2013.766929>
- Radford, K., Sutton, C., Sach, T., Holmes, J., Watkins, C., Forshaw, D., Jones, T., Hoffman, K., O'Connor, R., Tyerman, R., Merchán-Baeza, J. A., Morris, R., McManus, E., Drummond, A., Walker, M., Duley, L., Shakespeare, D., Hammond, A., & Phillips, J. (2018). Early, specialist vocational rehabilitation to facilitate return to work after traumatic brain injury: The FRESH feasibility RCT. *Health Technology Assessment*, *22*(33), 1-124. <https://doi.org/10.3310/hta22330>
- Rahja, M., Nguyen, K.-H., Laver, K., Clemson, L., Crotty, M., & Comans, T. (2020). Implementing an evidence-based dementia care program in the Australian health context: A cost–benefit analysis. *Health and Social Care in the Community*, *28*(6), 2013-2024. <https://doi.org/10.1111/hsc.13013>
- Rasmussen, R. S., Østergaard, A., Kjær, P., Skerris, A., Skou, C., Christoffersen, J., Seest, L. S., Poulsen, M. B., Rønholt, F., & Overgaard, K. (2016). Stroke rehabilitation at home before and after discharge reduced disability and improved quality of life: A randomised controlled trial. *Clinical Rehabilitation*, *30*(3), 225-236. <https://doi.org/10.1177/0269215515575165>
- Roderick, P., Low, J. F., Day, R., Peasgood, T., Mullee, M. A., Turnbull, J. C., Villar, T., & Raferty, J. (2001). Stroke rehabilitation after hospital discharge: A randomized trial comparing domiciliary and day-hospital care. *Age and Ageing*, *30*(4), 303-310. <https://doi.org/10.1093/ageing/30.4.303>
- Rodgers, H., Howel, D., Bhattarai, n., Cant, R., Drummond, A., Ford, G. A., Forster, A., Francis, R., Hills, K., Laverty, A. M., McKevitt, C., McMeekin, P., Price, C. I., Stamp, E., Stevens, E., Vale, L., & Shaw, L. (2019). Evaluation of an Extended Stroke Rehabilitation Service (EXTRAS). *Stroke*, *50*(12), 3561-3568. <https://doi.org/https://doi.org/10.1161/strokeaha.119.024876>
- Rodgers, H., Mackintosh, J., Price, C. I., Wood, R., McNamee, P., Fearon, T., Marritt, A., & Curless, R. (2003). Does an early increased-intensity interdisciplinary upper limb therapy programme following acute stroke improve outcome? *Clinical Rehabilitation*, *17*(6), 579-589. <https://doi.org/10.1191/0269215503cr652oa>
- Rogers, A. T., Bai, G., Lavin, R. A., & Anderson, G. F. (2016). Higher hospital spending on occupational therapy is associated with lower readmission rates. *Medical Care Research and Review*, *74*(6), 668-686. <https://doi.org/10.1177/1077558716666981>
- Ruchlin, H. S., Elkin, E. B., & Allegrante, J. P. (2001). The economic impact of a multifactorial intervention to improve postoperative rehabilitation of hip fracture patients. *Arthritis Care & Research*, *45*(5), 446-452. <https://doi.org/10.1002/1529-0131(200110)45:5><446::aid-art364>3.0.co;2-r
- Sackley, C. M., Walker, M. F., Burton, C. R., Watkins, C. L., Mant, J., Roalfe, A. K., Wheatley, K., Sheehan, B., Sharp, L., Stant, K. E., Fletcher-Smith, J., Steel, K., Barton, G. R., Irvine, L., & Peryer, G. (2016). An occupational therapy intervention for residents with stroke-related disabilities in UK Care Homes (OTCH): Cluster randomised controlled trial with economic evaluation. *Health Technology Assessment*, *20*(15), 1-138. <https://doi.org/https://doi.org/10.3310/hta20150>
- Sahota, O., Pulikottil-Jacob, R., Marshall, F., Montgomery, A., Tan, W., Sach, T., Logan, P. A., kendrick, D., Watson, A., Walker, M., & Waring, J. (2017). The community in-reach rehabilitation and care transition (CIRACT) clinical and cost-effectiveness randomisation controlled trial in older people admitted to hospital as an acute medical emergency. *Age and Ageing*, *46*(1), 26-32. <https://doi.org/https://doi.org/10.1093/ageing/afw149>
- Salkeld, G., Cumming, R. G., O'Neill, E., Thomas, M. R., Szonyi, G., & Westbury, C. (2000). The cost effectiveness of a home hazard reduction program to reduce falls among older persons. *Australian and New Zealand Journal of Public Health*, *24*(3), 265-271. <https://doi.org/10.1111/j.1467-842x.2000.tb01566.x>
- Sampson, C., James, M., Whitehead, P., & Drummond, A. (2014). An introduction to economic evaluation in occupational therapy: Cost-effectiveness of pre-discharge home visits after stroke (HOVIS). *British Journal of Occupational Therapy*, *77*(7), 330-335. <https://doi.org/10.4276/030802214X14044755581664>
- Samuelsson, K., & Wressle, E. (2014). Powered wheelchairs and scooters for outdoor mobility: A pilot study on costs and benefits. *Disability & Rehabilitation: Assistive Technology*, *9*(4), 330-334. <https://doi.org/10.3109/17483107.2013.827244>
- Schene, A. H., Koeter, M. W., Kikkert, M. J., Swinkels, J. A., & McCrone, P. (2007). Adjuvant occupational therapy for work-related major depression works: Randomized trial including economic evaluation. *Psychological Medicine*, *37*(3), 351-362. <https://doi.org/10.1017/S0033291706009366>
- Schneider, J., Duggan, S., Cordingley, L., Mozley, C. G., & Hart, C. (2007). Costs of occupational therapy in residential homes and its impact on service use. *Aging and Mental Health*, *11*(1), 108-114. <https://doi.org/https://doi.org/10.1080/13607860600963349>
- Shah, R. F., Zhang, S., Li, K., Baker, L., Sox-Harris, A., & Kamal, R. N. (2020). Physical and occupational therapy use and cost after common hand procedures. *Journal of Hand Surgery*, *45*(4), 289-297. <https://doi.org/https://doi.org/10.1016/j.jhsa.2019.09.008>
- Sheffield, C., Smith, C. A., & Becker, M. (2013). Evaluation of an agency-based occupational therapy intervention to facilitate aging in place. *Gerontologist*, *53*(6), 907-918. <https://doi.org/10.1093/geront/gns145>
- Shimada, T., Kobayashi, M., Ohori, M., Inagaki, Y., Shimooka, Y., & Ishihara, I. (2020). Cost-effectiveness of individualized occupational therapy for schizophrenia: Results from a two-year randomized controlled trial. *Asian Journal of Occupational Therapy*, *16*(1), 29-34. <https://doi.org/https://doi.org/10.11596/asiajot.16.29>
- Sigurdsson, E., Siggeirsdottir, K., Jonsson Jr., H., Gudnason, V., Matthiasson, T., & Jonsson, B. Y. (2008). Early discharge and home intervention reduces unit costs after total hip replacement: Results of a cost analysis in a randomised study. *International Journal of Health Care Finance and Economics*, *8*(3), 181-192. <https://doi.org/10.1007/s10754-008-9036-0>
- Sletten, C. D., Kurklinsky, S., Chinburapa, V., & Ghazi, S. (2015). Economic analysis of a comprehensive pain rehabilitation program: A collaboration between Florida Blue and Mayo Clinic Florida. *Pain Medicine*, *16*(5), 898-904. <https://doi.org/10.1111/pme.12679>
- Smeets, R. J. E. M., Severens, J. L., Beelen, S., Vlaeyen, J. W., & Knottnerus, J. A. (2009). More is not always better: Cost-effectiveness analysis of combined, single behavioral and single physical rehabilitation programs for chronic low back pain. *European Journal of Pain*, *13*(1), 71-81. <https://doi.org/https://doi.org/10.1016/j.ejpain.2008.02.008>
- Spoelstra, S. L., Sikorskii, A., Gitlin, L. N., Schueller, M., Kline, M., & Szanton, S. L. (2019). Dissemination of the CAPABLE model of care in a Medicaid Waiver program to improve physical function. *Journal of the American Geriatrics Society*, *67*(2), 363-370. <https://doi.org/https://doi.org/10.1111/jgs.15713>
- Sritipsukho, P., Riewpaiboon, A., Chaiyawat, P., & Kulkantrakorn, K. (2010). Cost-effectiveness analysis of home rehabilitation programs for Thai stroke patients. *Journal of the Medical Association of Thailand*, *93*(Supplement 7), 5262-5270.
- Sturkenboom, I. H. W. M., Hendriks, J. C. M., Graff, M. J. L., Adang, E. M. M., Munneke, M., Nijhuis-van der Sanden, M. W. G., & Bloem, B. R. (2015). Economic evaluation of occupational therapy in Parkinson's disease: A randomized controlled trial. *Movement Disorders*, *30*(8), 1059-1067. <https://doi.org/10.1002/mds.26217>
- Szanton, S. L., Alfonso, Y. N., Leff, B., Guralnik, J., Wolff, J. L., Stockwell, I., Gitlin, L. N., & Bishai, D. (2018). Medicaid cost savings of a preventive home visit program for disabled older adults. *Journal of the American Geriatrics Society*, *66*(3), 614-620. <https://doi.org/10.1111/jgs.15143>
- Tam, A., Mac, S., Isaranuwatchai, W., & Bayley, M. (2019). Cost-effectiveness of a high-intensity rapid access outpatient stroke rehabilitation program. *Journal of Rehabilitation Research*, *42*(1), 56-62. <https://doi.org/10.1097/MRR.0000000000000327>
- Teng, J., Mayo, N. E., Latimer, E., Hanley, J., Wood-Dauphinee, S., Côté, R., & Scott, S. (2003). Costs and caregiver consequences of early supported discharge for stroke patients. *Stroke*, *34*(2), 528-536. <https://doi.org/10.1161/01.str.0000049767.14156.2c>
- To, T. M., Exuzides, A., Abbass, I. M., Patel, A. M., Ta, J. T., Surinach, A., Fuller, R. L. M., & Luo, J. (2022). Health care resource utilization and costs among individuals with vs without Huntington disease in a US population. *Journal of Managed Care & Specialty Pharmacy*, *28*(11), 1228-1239. <https://doi.org/10.18553/jmcp.2022.28.11.1228>
- Toida, M., & Takemura, S. (2002). [Cost-benefit analysis of community based rehabilitation program using willingness to pay measured by the contingent valuation method]. *Nihon Koshu Eisei Zasshi*, *49*(1), 29-40.
- Tousignant, M., Desrosiers, J., Tourigny, A., & Robichaud, L. (2005). Costs of a home-based rehabilitation program for older adults after lower limb orthopedic surgery: A pilot study. *Archives of Gerontology and Geriatrics*, *41*(1), 51-60. <https://doi.org/https://doi.org/10.1016/j.archger.2004.11.002>
- Tung, Y.-J., Lin, W.-C., Lee, L.-F., Lin, H.-M., Ho, C.-H., & Chou, W. (2021). Comparison of cost-effectiveness between inpatient and home-based post-acute care models for stroke rehabilitation in Taiwan. *International Journal of Environmental Research and Public Health*, *18*(8), 4129. <https://doi.org/https://doi.org/10.3390/ijerph18084129>
- Turner-Stokes, L., Poppleton, R., Williams, H., Schoewenaars, K., & Badwan, D. (2012). Using the UKROC dataset to make the case for resources to improve cost-efficiency in neurological rehabilitation. *Disability and Rehabilitation*, *34*(22), 1900-1906. <https://doi.org/https://doi.org/10.3109/09638288.2012.670042>
- van den Hout, W. B., de Buck, P. D. M., & Vliet Vlieland, T. P. M. (2007). Cost-utility analysis of a multidisciplinary job retention vocational rehabilitation program in patients with chronic arthritis at risk of job loss. *Arthritis and Rheumatism*, *57*(5), 778-786. <https://doi.org/https://doi.org/10.1002/art.22786>
- van Meijeren-Pont, W., tamminga, S. J., Goossens, P. H., Groeneveld, I. F., Arwert, H., Meesters, J. J. L., Mishre, R. R., Vlieland, T. P. M., van den Hout, W. B., & group, T. S. C. O. o. R. S. s. (2021). Societal burden of stroke rehabilitation: Costs and health outcomes after admission to stroke rehabilitation. *Journal of Rehabilitation Medicine*, *53*(6), 00201. <https://doi.org/https://doi.org/10.2340/16501977-2829>
- Vincent, H. K., & Vincent, K. R. (2008). Functional and economic outcomes of cardiopulmonary patients: a preliminary comparison of the inpatient rehabilitation and skilled nursing facility environments. *American Journal of Physical Medicine & Rehabilitation*, *87*(5), 371-380. <https://doi.org/https://doi.org/10.1097/PHM.0b013e31816dd251>
- Visser, L. A., den Uijl, I., Redekop, W. K., Sunamura, M., Lenzen, M., Boersma, E., Brouwers, R. W. M., Kemps, H. M. C., van den Berg-Emons, H. J. G., & ter Hoeve, N. (2023). Cost-effectiveness of a cardiac rehabilitation program specifically designed for patients with obesity within the OPTICARE XL randomized controlled trial. *Archives of Physical Medicine & Rehabilitation*, *104*(6), 855-862. <https://doi.org/10.1016/j.apmr.2023.02.005>
- Von Koch, L., de Pedro-Cuesta, J., Kostulas, V., Almazán, J., & Widén Holmqvist, L. (2001). Randomized controlled trial of rehabilitation at home after stroke: One-year follow-up of patient outcome, resource use and cost. *Cerebrovascular Diseases*, *12*(2), 131-138. <https://doi.org/https://doi.org/10.1159/000047692>
- Vos-Vromans, D., Evers, S. M. A. A., Huijnen, I., Köke, A., Hitters, M., Rijnders, N., Pont, M., Knottnerus, A., & Smeets, R. (2017). Economic evaluation of multidisciplinary rehabilitation treatment versus cognitive behavioural therapy for patients with chronic fatigue syndrome: A randomized controlled trial. *PLoS One*, *12*(6), e0177260. <https://doi.org/https://doi.org/10.1371/journal.pone.0177260>
- Wales, K., Salkeld, G., Clemson, L., Lannin, N. A., Gitlin, L. N., Rubenstein, L. Z., Howard, K., Howell, M., & Cameron, I. D. (2018). A trial based economic evaluation of occupational therapy discharge planning for older adults: The HOME randomized trial. *Clinical Rehabilitation*, *32*(7), 919-929. <https://doi.org/https://doi.org/10.1177/0269215518764249>
- Wijnen, B. F. M., Hemmen, B., Bouman, A. I. E., van de Meent, H., Ambergen, T., Brink, P. R. G., Seelen, H. A. M., & Evers, S. M. A. A. (2019). Cost-effectiveness of an integrated ‘fast track’ rehabilitation service for multi-trauma patients: A non-randomized clinical trial in the Netherlands. *PLoS One*, *14*(3), e0213980. <https://doi.org/https://doi.org/10.1371/journal.pone.0213980>
- Williamson, K., Blane, D. N., Grieve, E., & Lean, M. E. J. (2023). Overlooked and under-evidenced: Community health and long-term care service needs, utilization, and costs incurred by people with severe obesity. *Clinical Obesity*, *13*(2), e12570. <https://doi.org/10.1111/cob.12570>
- Xie, F., Yan, J., Agarwal, G., & Ferron, R. (2021). Economic analysis of mobile integrated health care delivered by emergency medical services paramedic teams. *JAMA Network Open*, *4*(2), e21055. <https://doi.org/10.1001/jamanetworkopen.2021.0055>
- Yoshida, I., Hirao, K., & Kobayashi, R. (2019). The effect on subjective quality of life of occupational therapy based on adjusting the challenge-skill balance: A randomized controlled trial. *Clinical Rehabilitation*, *33*(1), 1732-1746. <https://doi.org/10.1177/0269215519858713>
- Yu, C. M., Lau, C. P., Chau, J., McGhee, S., Kong, S. L., Cheung, B. M. Y., & Li, L. S. W. (2004). A short course of cardiac rehabilitation program is highly cost effective in improving long-term quality of life in patients with recent myocardial infarction or percutaneous coronary intervention. *Archives of Physical Medicine and Rehabilitation*, *85*(12), 1915-1922. <https://doi.org/https://doi.org/10.1016/j.apmr.2004.05.010>
- Zeidler, J., Mittendorf, T., G., W., Zeidler, H., & Merkesdal, S. (2008). Comparative cost analysis of outpatient and inpatient rehabilitation for musculoskeletal diseases in Germany. *Rheumatology*, *47*(10), 1527-1534. <https://doi.org/https://doi.org/10.1093/rheumatology/ken315>
- Zhao, M., Haley, D. R., Nolin, A. M., Dunning, K., Wang, J., & Sun, Q. (2009). Utilization, cost, payment, and patient satisfaction of rehabilitative services in Shandong, China. *Health Policy*, *93*(1), 21-26. <https://doi.org/https://doi.org/10.1016/j.healthpol.2009.05.011>
- Zingmark, M., Nilsson, I., Fisher, A. G., & Lindholm, L. (2016). Occupation-focused health promotion for well older people - A cost-effectiveness analysis. *British Journal of Occupational Therapy*, *79*(3), 153-162. <https://doi.org/10.1177/0308022615609623>
- Zingmark, M., Nilsson, I., Norström, F., Göran Sahlén, K., & Lindholm, L. (2017). Cost effectiveness of an intervention focused on reducing bathing disability. *European Journal of Ageing*, *14*(3), 233-241. <https://doi.org/10.1007/s10433-016-0404-1>
- Zorowitz, R. D., Chen, E., Bianchini Tong, K., & Laouri, M. (2009). Costs and rehabilitation use of stroke survivors: A retrospective study of Medicare beneficiaries. *Topics in Stroke Rehabilitation*, *16*(5), 309-320. <https://doi.org/10.1310/tsr1605-309>
